# Inhibitory Decay and Supercritical Brain Dynamics During Sleep Deprivation

**DOI:** 10.1002/advs.75698

**Published:** 2026-05-15

**Authors:** Dai Zhang, Liqin Zhou, Rong Wang, Yuehua Han, Zhentao Zuo, Yanghua Tian, Ke Zhou

**Affiliations:** ^1^ Department of Radiology The Second Affiliated Hospital of Anhui Medical University Hefei China; ^2^ Medical Imaging Research Center Anhui Medical University Hefei China; ^3^ Beijing Key Laboratory of Applied Experimental Psychology National Demonstration Center for Experimental Psychology Education (Beijing Normal University) Faculty of Psychology Beijing Normal University Beijing China; ^4^ State Key Laboratory of Cognitive Science and Mental Health Institute of Biophysics Chinese Academy of Sciences Beijing China; ^5^ Department of Neurology The First Affiliated Hospital of USTC Division of Life Sciences and Medicine University of Science and Technology of China Hefei China; ^6^ Department of Neurology the First Affiliated Hospital of Anhui Medical University Hefei China

**Keywords:** criticality, excitation–inhibition balance, inhibitory efficacy, neuronal avalanches, resting‐state fMRI, sleep deprivation

## Abstract

Sleep deprivation (SD) changes brain‐wide dynamics, but the circuit‐level perturbation that can generate this systems‐level shift remains unclear. We scanned 26 participants at seven time points across 36 h of continuous wakefulness and assessed criticality from resting‐state functional Magnetic Resonance Imaging (rs‐fMRI) blood‐oxygen‐level‐dependent (BOLD) signals using neuronal avalanche metrics (branching ratio and mean avalanche size). The branching ratio increased from 0.98 at baseline to 1.08 after 36 h, indicating a progressive shift from near‐critical to supercritical propagation. Interestingly, the shift was heterogeneous. Visual and sensorimotor networks showed the largest deviations, whereas the limbic network remained close to criticality. Criticality changes tracked accumulated subjective sleep pressure but were largely dissociated from psychomotor vigilance lapses. SD also reshaped functional network organization, with the functional connectivity (FC) degree distribution shifting toward more high‐degree nodes. In a recurrent excitatory–inhibitory network model, gamma‐band power provided an interpretable proxy for effective gain and inhibitory control. Using this proxy, selectively reducing inhibitory efficacy was sufficient to capture the direction of the near‐critical‐to‐supercritical drift and a limbic‐like resilience pattern, supporting inhibitory decay as a plausible candidate circuit‐level mechanism linking SD to large‐scale propagation instability.

## Graphical abstract

Sleep deprivation progressively shifts human brain dynamics from near‐critical toward supercritical states, as revealed by neuronal avalanche analysis of resting‐state fMRI. These changes track subjective sleep pressure rather than vigilance lapses and show marked network heterogeneity. A circuit model suggests that reduced inhibitory efficacy provides a plausible mechanism linking accumulated sleep pressure to large‐scale propagation instability.

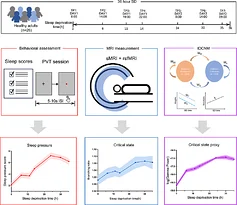

## 1 Introduction

Sleep deprivation (SD) is increasingly common and reliably impairs vigilance and cognitive abilities [1, 2, 3, 4]. Beyond gradual performance declines, SD can trigger state‐like events in the awake brain. A recent multimodal study showed that attentional failures after SD are time‐locked to coordinated neurovascular, pupillary, and CSF flow dynamics, consistent with transient shifts in arousal state [5]. At the circuit level, prolonged wakefulness has been linked to systematic changes in human cortical excitability [6] and to altered synaptic plasticity consistent with disrupted homeostasis [7]. Sleep loss can also drive broad changes in excitatory and inhibitory synaptic markers in animal models [8]. Together, these findings motivate a mechanistic account that links local excitation–inhibition regulation to SD‐related changes in large‐scale brain dynamics. What remains missing is a computationally interpretable mechanism that links SD‐related circuit changes to shifts in large‐scale propagation and stability in humans, especially one that can be tested at the level of large‐scale networks.

Resting‐state functional magnetic resonance imaging (rs‐fMRI) provides a noninvasive window into large‐scale coordination without task demands [9, 10]. rs‐fMRI studies of SD have found widespread alternations in functional connectivity (FC) within and between visual, sensorimotor, default mode, central executive, and dorsal attention networks, as well as disrupted interactions among large‐scale networks that relate to individual vulnerability to sleep loss [11, 12, 13, 14, 15, 16]. However, rs‐fMRI analyses typically summarize correlation‐based FC, e.g., the connectivity matrix itself, within‐ or between‐network averages, or derived graph‐theoretic measures, which characterizes statistical dependence rather than an explicit generative or mechanistic account of interactions [17, 18, 19]. Therefore, these summaries do not directly quantify the brain's dynamical operating regime, e.g., propagation stability, nor its time‐resolved evolution with time awake and across networks, motivating complementary dynamical readouts [20].

A natural dynamical framework for quantifying propagation stability is criticality [21, 22, 23]. Empirical and theoretical work has suggested that healthy brain activity often operates near a transition between order and disorder, although the strength and interpretation of the evidence remain actively debated [22, 23, 24]. Critical dynamics have been proposed to support efficient information transmission and integration, as well as maximum dynamic range [25, 26]. A common operationalization of criticality is the critical branching process. The branching ratio σ summarizes whether propagation tends to fade out (σ < 1), remain approximately sustained (σ ≈ 1), or amplify (σ > 1). In the classic critical branching view, σ ≈ 1 marks the boundary between decay and runaway amplification and is closely linked to scale‐invariant avalanche statistics [25]. Neuronal avalanche statistics provide an operational way to estimate these propagation properties from population activity, including macroscopic recordings [27, 28], and have been used to probe deviations from criticality across neurological and psychiatric disorders, including epilepsy and major depressive disorder [29, 30].

Despite these advances, the time‐resolved trajectory of human brain criticality during SD remains poorly characterized. Many studies contrast a single well‐rested session with one deprived session, leaving unresolved whether deviations accumulate progressively, whether they are uniform across functional networks, and whether some systems remain comparatively stable [11, 14]. This network‐level question is particularly important given evidence that criticality can be differentially modulated at the level of perception‐related cortical subsystems [31]. Moreover, SD combines a gradual build‐up of homeostatic sleep pressure with intermittent attentional lapses, which are often quantified by psychomotor vigilance measures [32, 33, 34]. Computational electroencephalogram (EEG) work suggests that inferred excitation–inhibition shifts accompany natural state fluctuations and vigilance impairment under SD [35], but how these processes map onto network‐resolved criticality and whether they coincide with systematic reorganization of functional network topology remains unclear.

Even when macroscopic signatures of altered criticality are robust, they do not identify the circuit changes that generate them. Local excitation–inhibition interactions provide a natural candidate mechanism because inhibitory control sets the gain and propagation of activity in recurrent networks [35, 36, 37]. This raises a mechanistic sufficiency question. In a recurrent excitation–inhibition model with a fixed coupling architecture, is selectively degrading inhibitory efficacy sufficient to produce the SD‐like drift from near‐critical toward supercritical propagation? Importantly, any plausible mechanism must also satisfy a network‐level constraint such that it should reproduce heterogeneous, network‐specific deviations while preserving the relative stability of networks that remain resilient.

Here, we address these questions by combining dense within‐subject sampling across 36 h of continuous wakefulness with neuronal avalanche analysis of rs‐fMRI. Rather than serving as a purely descriptive marker, the avalanche analysis provides a macroscale, system‐level readout of propagation stability that is directly interpretable within a criticality framework. We quantify this dynamical regime using the branching ratio and mean avalanche size at both the whole‐brain and canonical functional‐network levels, and relate the resulting trajectory to subjective sleep pressure and vigilance performance. In this way, the empirical analyses define the phenomenological target for mechanism building in a manner that the behavioral trajectory alone cannot, by revealing not only a progressive drift from near‐critical toward more supercritical dynamics during prolonged wakefulness, but also a key network‐level constraint that this shift is heterogeneous across functional systems. To move beyond association, we then perform a mechanistic sufficiency analysis using an Inhibitory‐Decay Cortical Network Model (IDCNM). Specifically, we test whether selectively degrading inhibitory efficacy is sufficient to account for both the global shift in propagation regime and the observed hierarchy of network susceptibility. By integrating longitudinal human imaging with a minimal, circuit‐interpretable model, this study uses macroscale avalanche dynamics to place top‐down constraints on candidate microcircuit mechanisms and to generate testable predictions about how SD destabilizes large‐scale brain propagation.

## 2 Materials and Methods

### 2.1 Participants

Volunteers were first selected from respondents to a web‐based questionnaire, all of them satisfied the following criteria: (1) nonsmokers and not alcoholics, (2) drank fewer than 3 caffeinated drinks daily, (3) not on any medications, (4) have no history of neurological or psychiatric disorders, (5) no symptoms associated with sleep disorders, (6) have habitually good sleeping habits (no insomnia, no excessive sleep), (7) normal or corrected‐to‐normal visual acuity, and (8) have no contraindications to MRI.

Each volunteer was invited to the laboratory and informed of the purpose and design of the study. They all signed a written informed consent approved by the Ethical Committee of the Institute of Biophysics, Chinese Academy of Sciences. Participants reported low‐to‐moderate habitual caffeine use (≤3 standard caffeinated drinks per day) and no history of stimulant misuse. This criterion was adopted to reduce withdrawal risk while enabling pre‐scan abstinence. All participants were given wrist actigraphy (Acti‐watch, Xiaomi, China) to objectively record their sleep and were asked to maintain a regular sleep schedule for one month (wake up between 6:00 and 9:00 and sleep 6.5 to 8.5 h per night). Participants with any abnormal data or irregular sleep patterns were excluded from the subsequent study. Records showed that they fell asleep between 23:13 and 00:53 and woke up between 6:30 and 8:46. The average sleep time per night was 7.6 ± 0.5 h. Anyone who did not follow the regular sleep schedule strictly was excluded from further investigation. 26 healthy participants (14 males, 12 females; aged 22.04 ± 2.46 years) were eventually participated in the present study. The participants in the present study were drawn from the same SD cohort reported in Ref. [38]. However, the current study addressed different research questions and focused on neuronal avalanche dynamics, criticality‐related measures, functional degree distributions, and computational model analyses that were independent of the findings reported previously.

### 2.2 SD Procedure

On Day 0, participants reported to the lab and were informed of the purpose and design of the study. Afterward, participants were assigned to pre‐arranged rooms for the night and maintained a normal sleep schedule without consuming caffeine. After reporting in and throughout the SD experiment, participants refrained from consuming caffeinated beverages or food. Therefore, no caffeine was consumed for at least 17 h before the first MRI scan and throughout the 36‐h SD period. This approach was consistent with previous studies [39, 40], and participants avoided caffeine intake for at least 12 h prior to being scanned. They were told to adhere to the regular sleep schedule to go to bed and get up. According to their actigraphy records, they fell asleep at around 23:50 and woke up at around 07:18. The average sleep duration was 7.5±0.5 h. Participants returned to the laboratory at 08:00 the following day (Day 1) and were then kept awake in the laboratory under continuous behavioral monitoring until 19:00 the next day (Day 2). Specifically, the participants stayed in a temperature‐controlled (24 ± 1°C) laboratory room with a fixed light level (<100 lux). They were allowed to drink water, eat chocolate‐free snacks (e.g., sandwiches, salads, rice, and vegetable rolls), and engage in nonstrenuous activities such as drawing, reading, and conversation. This was consistent with previous SD experiments [41, 42], aiming to minimize the impact of environmental factors on vigilance and physiological state. The experimenters monitored the participants continuously and gently woke them up when they were about to fall asleep. Simultaneous EEG or electrooculography (EOG) was not acquired in this protocol. Vigilance was monitored using eyes‐open fixation and continuous MR‐compatible video monitoring with operator intervention between runs if sustained eye closure or drowsiness was observed. The volunteers were not provided with information about the time during SD. The SD lasted for 36 h, during which the participants completed 9 times of rs‐fMRI measurements and behavioral assessments (Day 1: 08:00, 14:00, 19:00, and 22:00; Day 2: 01:00, 04:00, 08:00, 14:00, and 19:00, Figure 1A). After the ninth MRI scan, the volunteers took a 12‐h recovery sleep. Although nine rs‐fMRI sessions were acquired during the 36‐h SD protocol, the primary analyses focused on seven sessions (08:00, 14:00, 19:00, and 22:00 on Day 1; 08:00, 14:00, and 19:00 on Day 2). The 01:00 and 04:00 sessions were not excluded for data‐quality reasons. Rather, they were treated as nocturnal‐trough observations and were not included in the primary longitudinal analysis because the present analytic framework focused on the deprivation‐related trajectory across repeated daytime/evening measurements. As these two time points were isolated nocturnal measurements without corresponding reference points on the preceding day, their inclusion would have reduced comparability across time points and complicated longitudinal interpretation.

**FIGURE 1:**
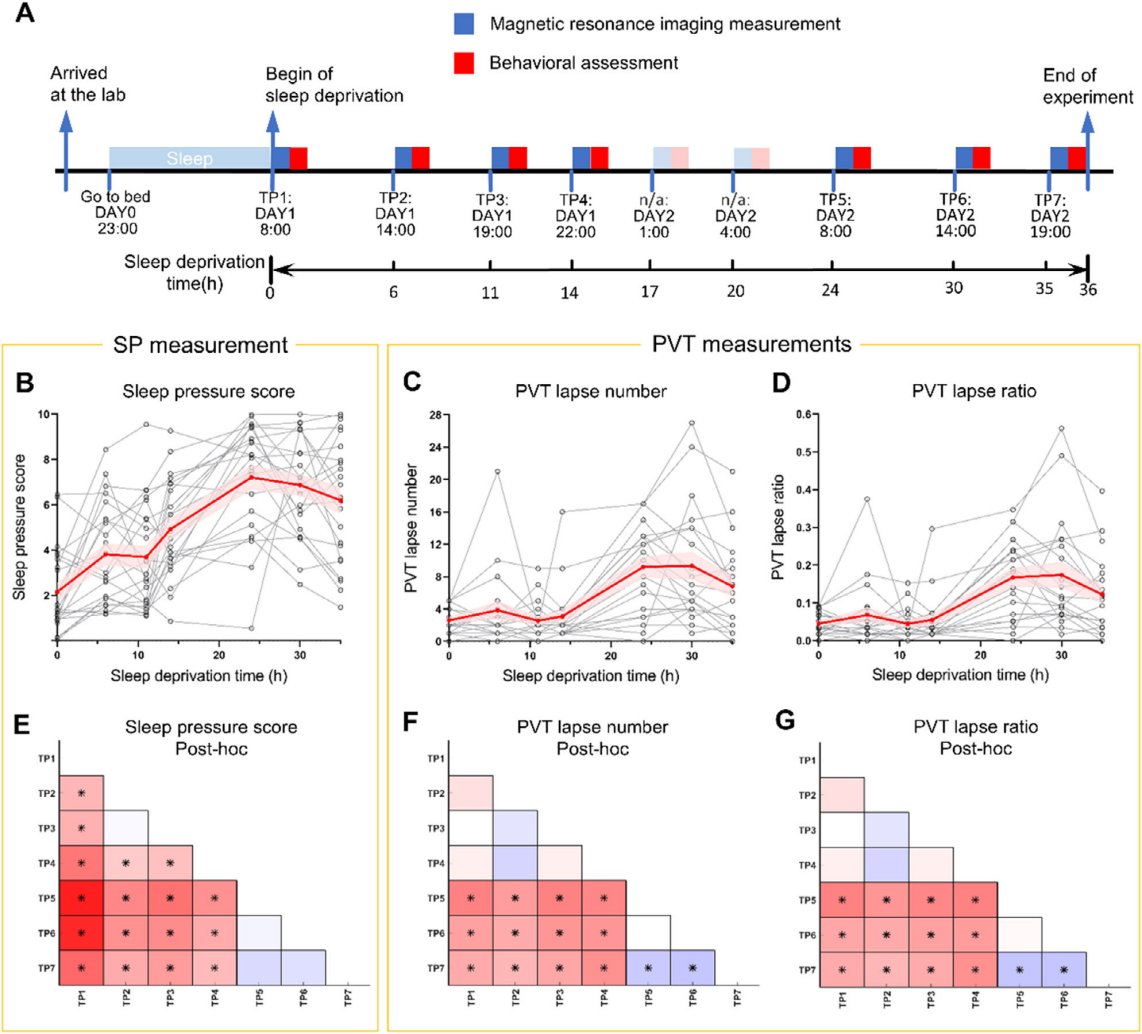
Sleep deprivation experimental design and behavioral measurements. (A) The night before sleep deprivation began, participants went to sleep at 11 p.m. in a hotel near the lab. During the 36‐h sleep deprivation, the participants underwent nine assessments, including MRI scans (marked by blue squares) and behavioral measures (marked by red squares). Data from two of these assessments were not included in this study and are marked with semi‐transparent squares. (B, C, D) The dynamic curves of sleep pressure scores, PVT lapse numbers, and PVT lapse ratio during the SD period are shown separately. The light red area represents SEM. (E, F, G,) Results of post‐hoc tests of sleep pressure score, PVT lapse numbers, and PVT lapse ratio are shown, respectively. Red indicates an increase in the behavioral measurement value at the second time point compared to the measurement value at the first time point, and blue indicates a decrease in the measurement value. Asterisks indicate a significant difference at *p* < 0.05 (FDR corrected). SP, sleep pressure; PVT, psychomotor vigilance task. Behavioral measures shown in panels B–D were reported previously in Zhang et al. [38] and are included here to provide context for the present analyses.

### 2.3 Behavioral Measurements

The behavioral measurements were conducted immediately after each MRI scan, including self‐assessment of sleep pressure and psychomotor vigilance task (PVT) performance. For the sleep pressure self‐assessment, each participant was asked to rate their own sleep pressure on a scale of 1 to 10, with higher scores indicating increased levels of sleep pressure. Because behavioral data were incomplete at a few sessions for some participants (e.g., brief device or software issues, time constraints between MRI blocks, or insufficient valid trials under pre‐specified quality thresholds), the sample size differed slightly across behavioral measures and time points. As a result, 23 participants completed all sleep pressure assessments. The PVT was employed to evaluate the participants’ vigilance and responsiveness [34, 43]. An advantage of the PVT is that it is almost unaffected by aptitude (inter‐individual variability) or learning (within‐subject variability) [32, 33]. In this task, the participants were required to press a button as soon as a red dot appeared in the center of the screen. Over the course of 6 min, the red dot emerged at random every few seconds and remained on the screen for five hundred milliseconds. If the participant did not respond within 500 ms, it was recorded as a lapse. The number and ratio of 20 participants’ lapses in responding to red dots were recorded in order to evaluate their sustained vigilance.

### 2.4 MRI Data Acquisition

MRI images were obtained on a 3T Siemens Prisma system (Siemens Healthcare, Erlangen, Germany) equipped with a 64‐channel head coil at the Beijing MRI Center for Brain Research of the Chinese Academy of Sciences. High spatial resolution T1 anatomical images were acquired using a 3D T1‐weighted, magnetization‐prepared rapid acquisition gradient‐echo sequence (time repetition [TR] = 2200 ms, time echo [TE] = 3.49 ms, time to inversion = 1000 ms, flip angle = 8°, slice thickness = 1.0 mm, in‐plane resolution = 1.0 mm × 1.0 mm, field of view = 256 mm × 224 mm). Whole‐brain functional images were obtained using a T2∗‐weighted gradient echo‐planar imaging sequence (GRE‐EPI, TR = 1000 ms, TE = 29 ms, flip angle = 40°, 65 slices, slice thickness = 2.0 mm, gap = 0 mm, in‐plane resolution = 2.0 mm × 2.0 mm, field of view = 192 mm × 192 mm, multi‐band factor = 5). During the resting state, the subjects were instructed to remain still and awake with their eyes open and focused on a fixation cross during the scan.

### 2.5 Rs‐fMRI Data Preprocessing

Rs‐fMRI data preprocessing was performed by AFNI (version 22.2.10) and FSL (version 6.0.5.2) software. After discarding the initial 5 volumes, the volumes were slice‐time corrected. The EPI volumes were then motion‐corrected with six rigid‐body transformation parameters, and followed by co‐registration to Montreal Neurological Institute (MNI) standard space with the anatomical scan. Rs‐fMRI from all participants across all time points met the criteria of maximum head displacement <3 mm and rotation <3 degrees. To enhance the signal‐to‐noise ratio (SNR) and minimize the effect of smoothness, EPI volumes were spatially smoothed with a 3.5‐mm full‐width half‐maximum Gaussian kernel and then intensity‐normalized by their mean signals. For each participant, white matter (WM) and cerebrospinal fluid (CSF) masks were generated using the “fsl_anat” pipeline in FSL and subsequently resampled to match the EPI resolution. To reduce non‐neuronal confounds, linear trends, head motion parameters, and the mean time series extracted from the WM and CSF masks were regressed out from the fMRI time series. Considering that frequencies outside the traditional 0.01–0.1 Hz range of rs‐fMRI signals also encoded meaningful neural information [44, 45], no temporal bandpass filtering was applied to the blood‐oxygen‐level‐dependent (BOLD) signals, particularly in the context of dynamic criticality within brain activities. A consistent gray matter mask was applied to each individual across all time points. Thus, in the subsequent neural avalanche analysis, data at each time point from all participants had the same number of voxels.

### 2.6 Neuronal Avalanche Analysis

**FIGURE 2:**
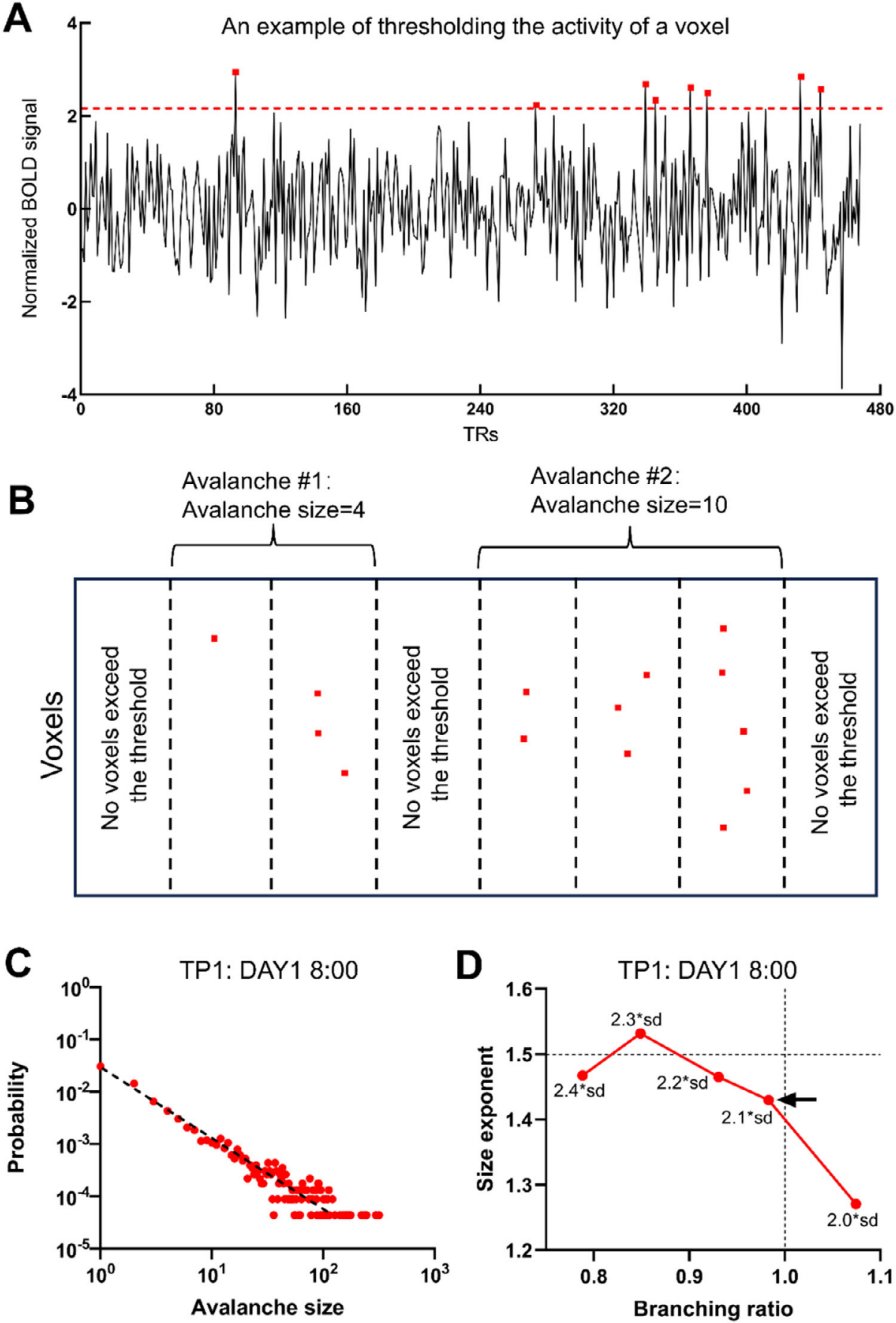
Neural avalanche analysis procedure. (A) The curve shows the standard normalized BOLD signal for a given voxel within the brain. A threshold (dashed red line) was applied to filter out the time series events (red squares). (B) Two avalanches are shown in the figure, both starting and ending with an empty time bin (all voxel activity does not exceed the threshold). The total number of events during the avalanche is the “avalanche size”. The two avalanches shown have sizes of 4 and 10. (C) When the threshold is 2.1 times the standard deviation, the power law distribution of the avalanche size at TP1 (Day 1 8:00) is shown. (D) At TP1 (Day 1 8:00), the size index and branching ratio of the avalanche size distribution change under different threshold parameters. The neural avalanche parameters resulting from the selected BOLD threshold should be closest to the intersection of the dashed lines (size exponents = 1.5, branching ratio = 1), marked by the black arrow.

In the present study, we used a neuronal avalanche framework to characterize alterations in resting‐state brain dynamics during SD using Matlab 2018a (The MathWorks, Inc.). The repetition time was used as the time‐bin width, and only positive peaks in BOLD signals were considered. Specifically, the BOLD time series from each voxel was z‐transformed by subtracting the mean and dividing by the standard deviation (Figure 2A). This normalization ensured that event detection was based on relative fluctuations within each voxel's own time series, rather than the raw amplitude of the BOLD signal, thereby reducing the influence of baseline shifts and session‐level amplitude differences across SD [30]. The z‐scored BOLD signals were subsequently binarized using a predetermined threshold: activities exceeding the threshold were labeled as 1 (event), while sub‐threshold activities were labeled as 0 (non‐event). Volumes containing at least one event were considered as active time bins. Neuronal avalanches were defined as sequences of contiguous active time bins bounded by at least one inactive time bin on either side (Figure 2B). The size of each avalanche was quantified as the total number of events (i.e., supra‐threshold activations) it contained. Given the small number of avalanches per rs‐fMRI scan (475 time points, yielding approximately 50–80 avalanches per scan), we aggregated avalanches across participants for each time point during the SD period to generate a group‐level distribution, rather than estimating individual‐level distributions (Figure 2C). Following Xin et al. [30], because each rs‐fMRI scan yielded a limited number of avalanches, and scale‐specific avalanche counts were sparse at the individual‐scan level, thresholds were selected at the group level rather than optimized separately for each participant [30]. This choice was made to ensure consistent event definition across participants before pooling avalanches within each time point and to improve the stability of group‐level estimation of avalanche statistics.

In the avalanche analysis, the selection of the activation threshold was guided by the theoretical relationship between power‐law exponents and branching ratios [46]. To determine an appropriate threshold at the whole‐brain level, we systematically evaluated a range of z‐score thresholds (2.0 to 2.4 standard deviations [SD], with 0.1 SD step), and chose the threshold that best met the standard theory, i.e., the power‐law exponent approached ‐1.5 and the branching ratio approached 1 based on the data from the first time point (Day 1: 08:00), when the brain was not yet affected by SD (Figure 2D). This threshold was then used for the calculation of avalanche dynamic parameters at all time points.

Threshold selection for network‐level avalanche analysis followed a similar approach. Although the same theoretical criteria were applied, the optimal thresholds varied across networks due to differences in system size, i.e., the number of voxels within each network. Larger networks typically required higher BOLD thresholds. The Yeo 7‐network parcellation of the brain [47] was applied for network‐level analysis, which includes the visual network (VN), somatomotor network (SMN), dorsal attention network (DAN), ventral attention network (VAN), limbic network (LN), frontoparietal network (FPN), and default mode network (DMN). Accordingly, network‐specific threshold ranges were identified as follows: VN, 1.4–1.8 SD; SMN, 1.2–1.6 SD; DAN, 1.4–1.8 SD; VAN, 1.2–1.6 SD; LN, 1.1–1.5 SD; FPN, 1.4–1.8 SD; and DMN, 1.5–1.9 SD. Similarly, we selected BOLD thresholds for each network based on data from the first time point (Day 1: 08:00) and then used this threshold to generalize to data from other time points for calculations.

To evaluate alterations in critical brain dynamics during SD, we quantified two canonical parameters of neuronal avalanche activity: the branching ratio and the mean avalanche size. In systems operating near criticality, the branching ratio is theoretically expected to approximate 1. Values greater than 1 indicate a supercritical regime, wherein activity tends to amplify over time, while values less than 1 suggest a subcritical regime characterized by diminishing activity propagation [48]. In this study, the branching ratio was computed for each participant as the average ratio of the number of suprathreshold events in a given active time bin to that in the immediately preceding bin, calculated across all active time bins. Additionally, to assess temporal changes in avalanche magnitude across the SD period, we calculated the mean avalanche size by averaging the number of events per avalanche across participants at each time point.

### 2.7 Correlation Between Criticality Parameters and Behavioral Measures

To assess the relationship between changes in criticality and behavioral measures during SD, we calculated Spearman's rank correlation coefficients between whole‐brain avalanche parameters (branching ratio, mean avalanche size) and behavioral measures (sleep pressure, PVT lapse number, and PVT lapse ratio) across all time points within each subject. Subsequently, the distributions of these correlation coefficients across all subjects (2 × 3 = 6 distributions of correlation coefficients in total) are shown separately. The distributions were compared to 0 using a one‐sample t‐test to assess the significance level. With consistent procedures, we also calculated the Spearman's rank correlation coefficients between behavioral indices and neuronal avalanche parameters for each network separately.

To further examine the longitudinal associations between avalanche dynamics and behavioral measures, we fitted a series of linear mixed‐effects models. Specifically, mean avalanche size and branching ratio were separately entered as dependent variables, while subjective sleep pressure, PVT lapse number, and PVT lapse ratio were each entered as fixed‐effect predictors in separate models, resulting in a total of six models. To obtain a more conservative estimate of these associations, SD duration (time awake) was additionally included as a fixed effect, and subject was modeled as a random intercept to account for within‐subject repeated measurements across the seven time points.

### 2.8 Functional Connectivity and Degree Distribution

FC was computed as the Pearson correlation between preprocessed BOLD time series for all gray‐matter voxels. Degree centrality was defined as the number of suprathreshold functional connections for each voxel in a binarized FC graph [49, 50]. Consistent with prior voxelwise degree‐centrality analyses, we restricted degree estimation to positive correlations and defined an edge as present when 𝑟 > 0.3 [51]. For each participant and time point, we summarized the voxelwise degree distribution and quantified the fraction of high‐degree nodes (degree > 300) and low‐degree nodes (degree < 30). Degree maps were visualized using BrainNet Viewer (http://www.nitrc.org/projects/bnv/).

### 2.9 Inhibitory‐Decay Cortical Network Modeling

**FIGURE 3:**
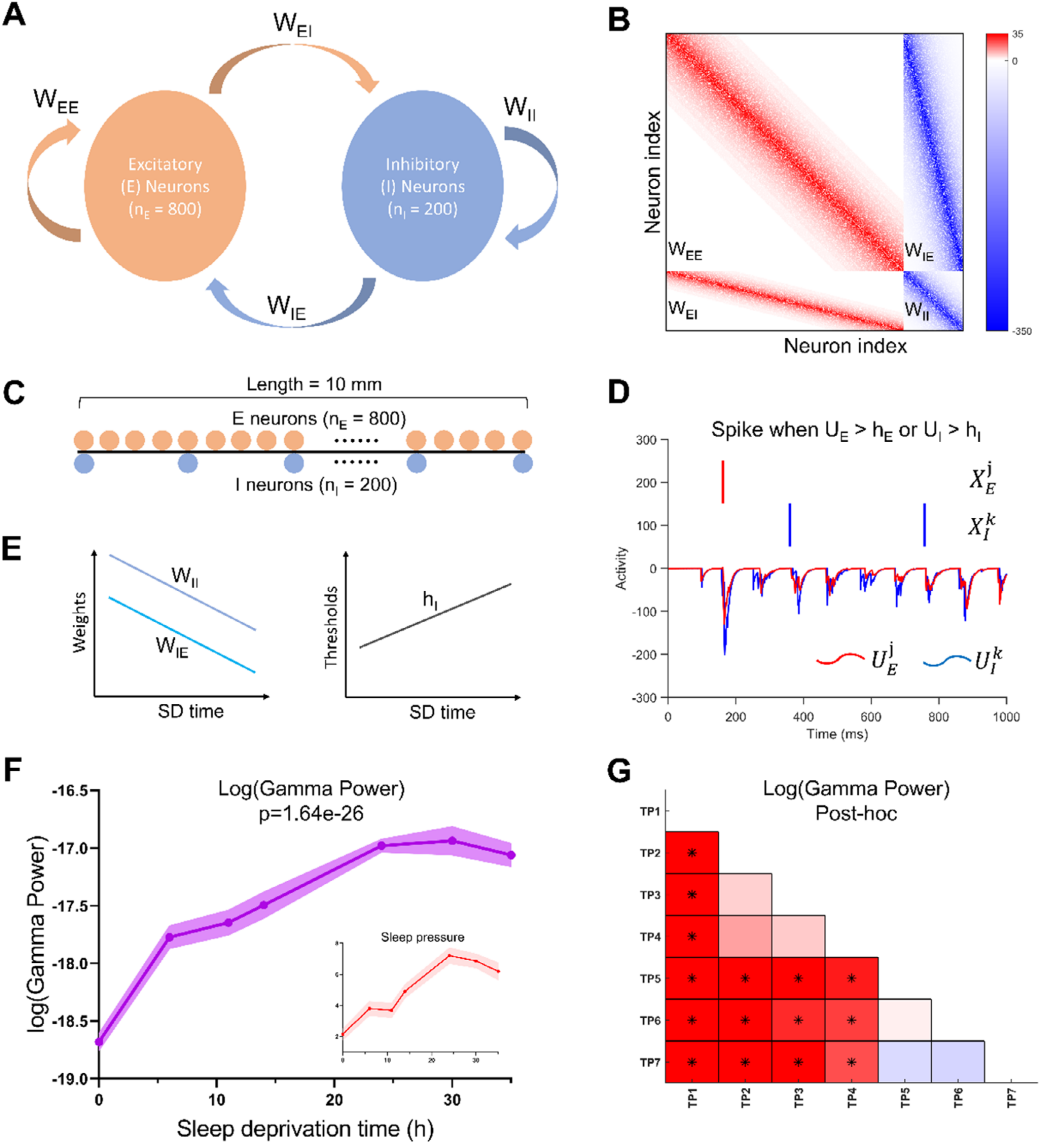
Sleep deprivation–driven degradation of inhibitory regulation reproduces supercritical dynamics in a recurrent cortical network model. (A) Schematic illustration of the recurrent cortical network model. The network consists of excitatory pyramidal neurons (E) and inhibitory interneurons (I) interacting through recurrent synaptic connections. Neuronal firing is governed by membrane potential dynamics, with excitation–inhibition balance determining the global dynamical regime of the network. (B) Synaptic connectivity matrix of the full network comprising 1 000 neurons. Each element represents the synaptic weight between a presynaptic and postsynaptic neuron, including E‐to‐E, E‐to‐I, I‐to‐E, and I‐to‐I connections. (C) Spatial organization of the network showing the distribution of 800 excitatory neurons (E) and 200 inhibitory neurons (I) along a one‐dimensional cortical domain. Neurons are uniformly distributed in space, allowing conduction delays to emerge naturally from inter‐neuronal distances. (D) Representative membrane potential traces and spike trains from one excitatory neuron and one inhibitory neuron under baseline (non–sleep‐deprived) conditions. Under the original parameter set, the network operates near a critical regime and exhibits spontaneous, irregular population activity with prominent low‐frequency oscillations. (E) Schematic illustration of sleep deprivation–induced parameter modulation in the computational model. Progressive sleep deprivation was simulated by a linear increase in the firing threshold of inhibitory neurons and a linear decrease in inhibitory synaptic weights, reflecting cumulative impairment of inhibitory efficacy with prolonged wakefulness. (F) Changes in simulated gamma‐band power (30–45 Hz) across different sleep deprivation durations. Gamma power was computed from a population‐level local field potential (LFP) proxy derived from the summed spiking activity of all 1 000 neurons and log‐transformed. Repeated‐measures ANOVA revealed a significant effect of sleep deprivation duration. Data are shown as mean ± SEM. (G) Post‐hoc comparisons of gamma power between time points. Gamma power was significantly elevated at early sleep deprivation stages compared with baseline, while later stages showed a tendency toward stabilization or slight decrease without reaching statistical significance. Asterisks indicate a significant difference at *p* < 0.05 (FDR corrected).

To understand the impact of SD on the brain's critical state from a computational perspective, we extended previous physiologically interpretable cortical network computational models [35, 52] by constructing a recurrent neural network model (IDCNM) with parameters modulated by SD (Figure 3A,B). We first introduce the original computational model, followed by the effect of SD on the model parameters.

The original model consists of two types of neurons: pyramidal excitatory neurons (E) and inhibitory interneurons (I). The ratio of the two types of neurons was set at 4:1, including *N_E_* excitatory neurons and *N_I_* inhibitory neurons, consistent with the common cell ratio characteristics in cortical tissue [52]. The two types of neurons are uniformly distributed within a one‐dimensional spatial domain with length *w* (Figure 3C). The firing of each neuron was modeled as a non‐homogeneous Poisson process, with its instantaneous firing probability determined by the neuron's membrane potential.

(1) $$\begin{array}{c} X_{E,I}^{j}=\sum \delta_{E,I}^{j}\;\left(t-t_{i}\right) \end{array}$$

where $X_{E,I}^{j}$ is the spike train of the *j* ^th^ neuron in the population E or I. Unit impulse function represented that an excitatory or inhibitory neuron fired at time *t_i_* when the membrane potential *u_E_* > *h_E_* or *u_I_* > *h_I_*.

The evolution of the membrane potential of neurons $u_{E}^{j}\left(t\right)$ and $u_{I}^{j}\left(t\right)$ is described by stochastic differential equations, which were set as follows.

(2) $$\begin{array}{ccc} \alpha_{E}^{-1}\;\frac{du_{E}^{j}\left(t\right)}{dt} & = & -u_{E}^{j}\left(t\right)+bv_{E}^{j}\left(t\right)+G_{EE}^{j}\left(t\right)+G_{IE}^{j}\left(t\right)+\sqrt{2D}\varphi_{E}^{j}\left(t\right)\;\\ j & = & 1,\text{…},N_{E} \end{array}$$

(3) $$\begin{array}{ccc} \alpha_{I}^{-1}\;\frac{du_{I}^{j}\left(t\right)}{dt} & = & -u_{I}^{j}\left(t\right)+bv_{I}^{j}\left(t\right)+G_{EI}^{j}\left(t\right)+G_{II}^{j}\left(t\right)+\sqrt{2D}\varphi_{I}^{j}\left(t\right)\;\\ j & = & 1,\text{…},N_{I} \end{array}$$

(4) $$\begin{array}{c} a^{-1}\;\frac{dv_{E}^{j}\left(t\right)}{dt}=-v_{E}^{j}\left(t\right)+u_{E}^{j}\left(t\right)\;\;j=1,\text{…},N_{E} \end{array}$$

(5) $$\begin{array}{c} a^{-1}\;\frac{dv_{I}^{j}\left(t\right)}{dt}=-v_{I}^{j}\left(t\right)+u_{I}^{j}\left(t\right)\;\;j=1,\text{…},N_{I} \end{array}$$

where $v_{E}^{j}\left(t\right)$ and $v_{I}^{j}\left(t\right)$ present adaptation currents with gain *b* and rate *a*. The $G_{NM}^{j}\left(t\right)$ presents cross‐population recurrent inputs, which are defined as follows:

(6) $$\begin{array}{c} G_{EE}^{j}\;\left(t\right)=\sum_{k=1}^{N_{E}}W_{EE}^{jk}\cdot EPSP^{k}\left(t-\tau^{jk}\right)\; \end{array}$$

(7) $$\begin{array}{c} G_{IE}^{j}\;\left(t\right)=\sum_{k=1}^{N_{I}}W_{IE}^{jk}\cdot IPSP^{k}\left(t-\tau^{jk}\right)\; \end{array}$$

(8) $$\begin{array}{c} G_{EI}^{j}\;\left(t\right)=\sum_{k=1}^{N_{E}}W_{EI}^{jk}\cdot EPSP^{k}\left(t-\tau^{jk}\right)\; \end{array}$$

(9) $$\begin{array}{c} G_{II}^{j}\;\left(t\right)=\sum_{k=1}^{N_{I}}W_{II}^{jk}\cdot IPSP^{k}\left(t-\tau^{jk}\right)\; \end{array}$$

where *EPSP^k^* (*t*) and *IPSP^k^* (*t*) present afferent postsynaptic excitatory and inhibitory potentials with time constant τ_*m*_:

(10) $$\begin{array}{c} EPSP^{k}\;\left(t\right)=\int_{0}^{t}X_{E}^{j}\left(s\right)\frac{1}{\tau_{m}}e^{-\frac{\left(t-s\right)}{\tau_{m}}}\;ds \end{array}$$

(11) $$\begin{array}{c} IPSP^{k}\;\left(t\right)=\int_{0}^{t}X_{I}^{j}\left(s\right)\frac{1}{\tau_{m}}e^{-\frac{\left(t-s\right)}{\tau_{m}}}\;ds \end{array}$$

The synaptic weights were set as follows:

(12) $$\begin{array}{c} W_{NM}^{jk}\;\left(c\right)=w_{NM}^{o}\;\left(c\right)e^{-\sigma_{N,M}^{2}\cdot \left|x\left(j\right)-x\left(k\right)\right|} \end{array}$$

where *N*, *M* represented E or I type. It should be noted that *x*(*j*) present spatial location of neuron *j* in the network space. Therefore, the propagation delays between neuron *j* and *k* could be calculated as τ^*jk*^ = |*x*(*j*) − *x*(*k*)|/*v* , with *v* being the axonal conduction velocity. If *N* represented E type, $\sigma_{N,M}^{2}=\sigma_{E}^{2}\;$, while if *N* represented I type, $\sigma_{N,M}^{2}=\sigma_{I}^{2}\;$. To represent the sparseness of cortical connections, synaptic weights $W_{NM}^{jk}\left(c\right)$ are set to 0 with probability of 1‐*c*. The parameter values in the original computational model are shown in Table 1. Previous studies have shown that this set of parameters puts the model in a critical state (Figure 3D) [35, 52]. We therefore treat it as a simulation without the effects of SD.

**TABLE 1:** IDCNM model parameters.

Symbol	Value	Symbol	Value
*N_E_*	800	$w_{EE}^{o}$	35
*N_I_*	200	$w_{EI}^{o}$	35
*h_E_*	0	$w_{IE}^{o}$	−350
*h_I_*	0	$w_{II}^{o}$	−350
*W*	10 mm	$\sigma_{E}^{2}$	1.0
α_*E*_	1.0	$\sigma_{I}^{2}$	0.5
α_*I*_	1.5	*v*	0.15 m/s
*a*	0.01	*D*	0.001
*b*	0.01	*dt*	1 ms
*C*	0.8	θ_*h*_	0.01
θ_*IE*_	0.01	θ_*II*_	0.01

IDCNM = inhibitory‐decay cortical network model.

Studies have shown that increased sleep pressure impairs the recruitment of GABAergic interneurons [53, 54]. Additionally, there was evidence supporting that SD primarily affected cortical inhibition [55, 56]. Taking all factors into account, we simulated the effects of SD by increasing the firing threshold of inhibitory neurons (*h_I_*) and decreasing the weight of inhibitory connections ($W_{IE}^{jk}$, $W_{II}^{jk})$. The linear scaling of both parameters with deprivation duration was adopted as a first‐order approximation (Figure 3E), capturing the progressive nature of sleep pressure accumulation and its impact on inhibitory circuits. Importantly, the magnitude of these adjustments was chosen to remain within a physiologically plausible range, ensuring that network dynamics were preserved and preventing artificial transitions to pathological states.

(13) $$\begin{array}{c} h_{I}\;\left(t\right)=h_{I}+\theta_{h}\cdot SD_{time} \end{array}$$

(14) $$\begin{array}{c} W_{IE}^{jk}\;\left(t\right)=W_{IE}^{jk}\;\cdot \left(1-\theta_{IE}\cdot SD_{time}\right) \end{array}$$

(15) $$\begin{array}{c} W_{II}^{jk}\;\left(t\right)=W_{II}^{jk}\;\cdot \left(1-\theta_{II}\cdot SD_{time}\right) \end{array}$$

Specifically, inhibitory synaptic weights were reduced by 1% per hour of SD, while the firing threshold of inhibitory neurons was increased by 0.01 mV per hour. These parameter changes were designed to reflect a slow, cumulative degradation of inhibitory efficacy rather than abrupt network destabilization [57].

### 2.10 Gamma Power Calculating

We first aggregated the spiking activity of all 1000 neurons in the RNN to obtain a population‐level measure of network firing. A local field potential (LFP) proxy was then constructed from the summed spike trains to capture collective neuronal dynamics. Gamma‐band power (30–45 Hz) was subsequently estimated from this LFP proxy using spectral analysis and log‐transformed to stabilize variance [35]. The resulting gamma power was used as an index of network gain, reflecting changes in excitation–inhibition balance and shifts in the network's operating regime relative to criticality [35].

### 2.11 Statistical Analyses

To examine changes in branching ratios, average avalanche sizes, node properties (i.e., proportions of high‐ and low‐degree nodes), and log‐transformed gamma power across the SD period, one‐way repeated‐measures analysis of variance (ANOVA) was conducted. Where applicable, post‐hoc comparisons were performed, and the false discovery rate was controlled using the Benjamini–Hochberg (FDR‐BH) correction for multiple comparisons. One‐sample t‐tests were employed to assess the significance of correlations between neuronal avalanche parameters and behavioral measures. Effect sizes for repeated‐measures analyses were quantified using partial eta squared (ηp^2^), as provided by SPSS, given its suitability for within‐subject designs. All statistical tests were two‐tailed, with a significance threshold set at *p* < 0.05. Data are presented as mean ± standard error of the mean (SEM). All statistical analyses were conducted using SPSS 22.0 (International Business Machines Corp.) and MATLAB 2018a.

### 2.12 Sensitivity Analysis

To assess the robustness of preprocessing choices, we repeated the whole‐brain avalanche analyses using a 4‐mm FWHM Gaussian smoothing kernel. In addition, we repeated the analyses under two temporal filtering conditions, including conventional band‐pass filtering at 0.01–0.1 Hz and isolation of frequency components above 0.1 Hz. The corresponding results are presented in (Figures S2 and S3).

In addition, to assess the robustness of the supercriticality results to threshold selection, supplementary analyses were performed using alternative fixed z‐score thresholds (z = 2.0 and z = 2.2), and the corresponding results are presented in (Figure S4). To assess whether the cross‐network heterogeneity of avalanche dynamics depended on network‐specific threshold calibration, supplementary analyses were performed using a common z‐score threshold (1.3 SD) across different networks, and the corresponding results are presented in (Figure S6). Further, robustness analyses were performed for the FC topology measures by systematically varying both the correlation threshold for functional connections and the degree cutoffs for defining high‐ and low‐degree nodes, and the detailed results are presented in (Figure S8).

## 3 Results

### 3.1 Sleep Pressure Rises Before Lapses

Behavioral measures are shown in Figure 1B–D. Behavioral changes in this cohort were reported previously in [38] and are included here to provide context for the present analyses. Both subjective sleep pressure (*F*_(6,132)_ = 27.02, *p* = 7.68e‐21, ηp^2^ = 0.55) and vigilance measures (PVT lapse number, *F*_(6,114)_ = 16.44, *p* = 1.42e‐13, ηp^2^ = 0.46; PVT lapse ratio, *F*_(6,114)_ = 15.86, *p* = 3.80e‐13, ηp^2^ = 0.46) changed significantly during SD. Both measures increased overall from Day 1 (08:00) to Day 2 (08:00) and then decreased later on Day 2 (14:00 to 19:00). However, their trajectories differed in timing. Post‐hoc tests showed that the sleep pressure at TP2 (Day 1: 14:00), TP3 (Day 1: 19:00), and TP4 (Day 1: 22:00) was significantly higher than TP1 (Day 1: 08:00, FDR‐corrected *p* < 0.05), whereas neither PVT lapse number nor lapse ratio differed from TP1 at those time points (Figure 1E–G). Conversely, both vigilance measures decreased significantly from TP6 to TP7 and from TP5 to TP7 (FDR‐corrected *ps* < 0.05), while sleep pressure showed only a non‐significant numerical decrease over the same intervals. Together, these results indicate that, in our protocol, subjective sleep pressure rose earlier than measurable changes in PVT lapses during SD.

### 3.2 Whole‐Brain Drift to Supercriticality

**FIGURE 4:**
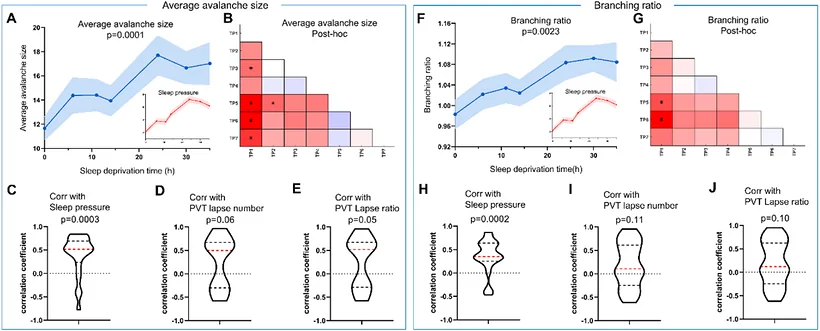
Changes of neural avalanche parameters during sleep deprivation. (A) The curve of average avalanche size during sleep deprivation. The *p*‐value was shown above the curve. For comparison purposes, the sleep pressure change curve is shown in the lower right corner of the figure, which is consistent with that in Figure 1B. (B) Results of post‐hoc tests of average avalanche size are shown. (C, D, E) The distribution of correlation between the average avalanche size and the sleep pressure, PVT lapse number, and PVT lapse ratio was shown, respectively. A one‐sample t‐test was used to compare the distribution to 0. Equivalent to the formats of (A, B), the curve of branching ratio during sleep deprivation (F) and the results of post‐hoc tests of branching ratio (G) were shown. Equivalent to the formats of (C–E), the correlation between the branching ratio and the sleep pressure (H), PVT lapse number (I), and PVT lapse ratio (J) was shown, respectively. For post‐hoc results, red indicates an increase in the behavioral measurement value at the second time point compared to the measurement value at the first time point, and blue indicates a decrease in the measurement value. Asterisks indicate a significant difference at *p* < 0.05 (FDR corrected).

To quantify SD‐related changes in large‐scale propagation dynamics, we computed whole‐brain neuronal avalanche metrics (i.e., branching ratio and mean avalanche size) from rs‐fMRI BOLD signals at each time point, using a fixed threshold of 2.1 SD, which yielded baseline dynamics close to criticality at TP1 (σ ≈ 1; see Methods). Both mean avalanche size (*F*_(6,132)_ = 5.95, *p* = 0.0001, ηp^2^ = 0.19) and branching ratio (*F*_(6,132)_ = 3.60, *p* = 0.0023, ηp^2^ = 0.13) increased significantly during SD (Figure 4A,F). The mean branching ratio rose from 0.98 at baseline (TP1) to 1.08 at TP7 (36 h), consistent with a progressive drift from critical (or near‐critical) dynamics (σ ≈ 1) toward a more supercritical regime (σ > 1).

Post‐hoc analyses showed that mean avalanche size was already significantly higher on the first day (TP3, Day 1: 19:00) than at TP1 (FDR‐corrected *p* < 0.05), accompanied by a numerical increase in branching ratio from 0.98 (TP1) to 1.04 (TP3). After the subsequent overnight wakefulness, both branching ratio and mean avalanche size were significantly elevated relative to earlier time points (FDR‐corrected *ps* < 0.05). No significant differences were observed between TP6 and TP7 for either metric (Figure 4B,G). These analyses indicate that whole‐brain avalanche metrics increased early during SD, rose further after overnight wakefulness, and then plateaued between TP6 and TP7.

### 3.3 Network Drift and Degree Distribution Shifts

**FIGURE 5:**
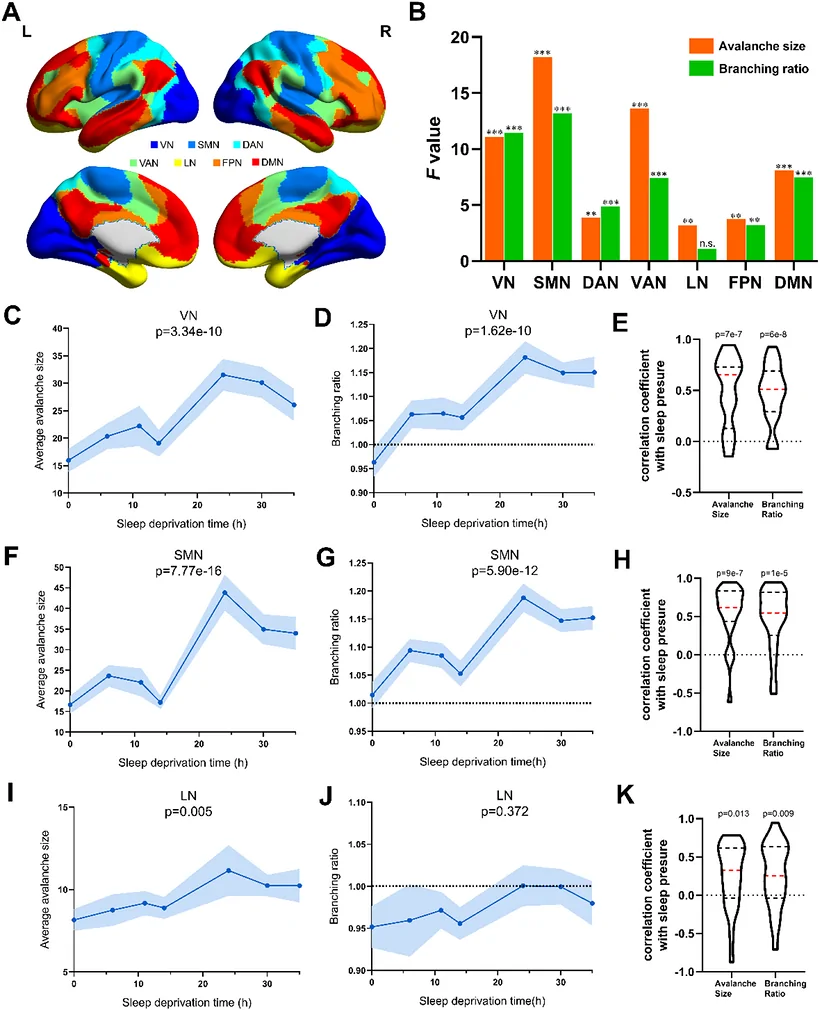
Network‐level avalanche parameters during sleep deprivation. (A) The Yeo 7‐network parcellation of the brain includes VN, SMN, DAN, VAN, LN, FPN, and DMN, which are marked with different colors. (B) *F* values of repeated measures ANOVA for average avalanche size and branching ratio among different network parcellations across 7 time points. Statistically significant points were labeled (^*^ *p* < 0.05; ^**^ *p* < 0.01; ^***^ *p* < 0.001). (C, D) The dynamic curves of the average avalanche size and branching ratio of VN during the SD period. The *p*‐value was shown above the curve. The case where the branching ratio is equal to 1 is shown by a dotted line, indicating the standard critical state. (E) The distribution of correlation coefficients between individuals’ sleep pressure and the average avalanche size and branching ratio of VN. A one‐sample t‐test was used to compare the distribution to 0. Equivalent to the formats of (C, D), the dynamic curves of the average avalanche size and branching ratio of SMN (F, G) and LN (I, J) were shown. Equivalent to the formats of (E), the distribution of correlation coefficients between individuals’ sleep pressure and the average avalanche size and branching ratio of SMN (H) and VN (K) were shown. VN, visual network; SMN, sensorimotor network; DAN, dorsal‐attention network; VAN, ventral‐attention network; LN, limbic network; FPN, frontoparietal network.

We next examined avalanche metrics within seven canonical functional networks (Figure 5A). Using network‐specific event thresholds (Table S1; see Methods), neuronal avalanche metrics changed heterogeneously across networks during SD (Figure 5B). The VN and SMN showed robust increases in branching ratio (VN: *F*_(6,150)_ = 11.44, *p* = 1.62e‐10, ηp^2^ = 0.31, Figure 5D; SMN: *F*_(6,150)_ = 13.18, *p* = 5.90e‐12, ηp^2^ = 0.35, Figure 5G). The branching ratios of VN (1.18) and SMN (1.19) reached maxima at TP5. In contrast, the LN showed no significant change in branching ratio (LN: *F*_(6,150)_ = 1.09, *P* = 0.372, ηp^2^ = 0.04, Figure 5J), remaining close to 1.0. This heterogeneity indicates that SD does not induce a uniform global gain change but instead reshapes propagation regimes in a network‐dependent manner. The remaining networks (DAN, VAN, FPN, and DMN) also exhibited significant increases (Figure S1). Mean avalanche size changes broadly mirrored branching ratio changes across networks (Figure 5C,F,I and Figure S1).

**FIGURE 6:**
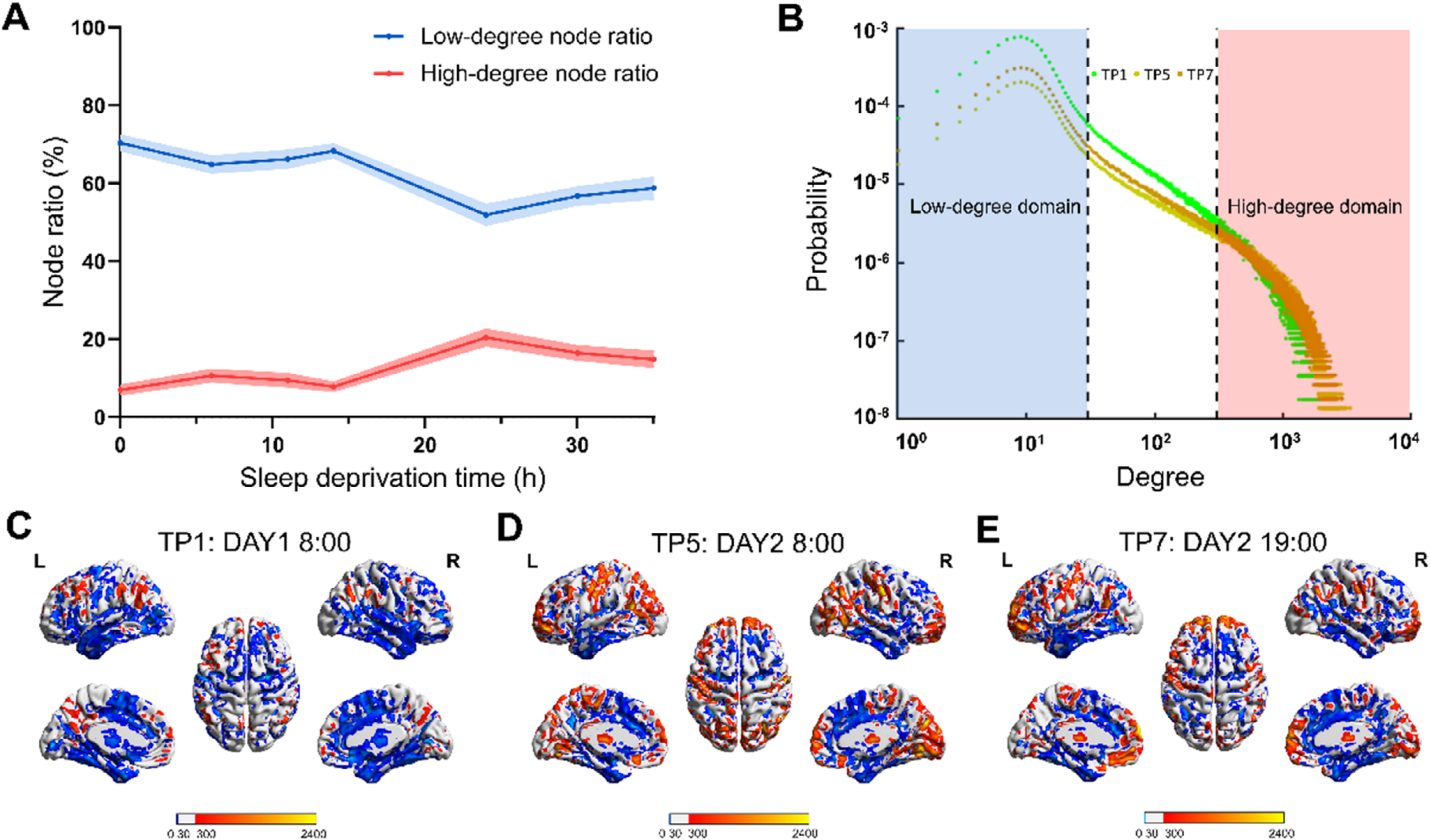
Functional connectivity changes during sleep deprivation. (A) The dynamic curves of low‐degree node ratio and high‐degree node ratio during the SD period are shown. Low‐degree nodes are defined as voxels with less than 30 whole‐brain functional connections (*r* > 0.3), and high‐degree nodes are defined as voxels with more than 300 whole‐brain functional connections (*r* > 0.3). (B) Degree distribution of whole‐brain voxels at three representative time points (TP1:DAY1 8:00; TP5: DAY2 8:00; TP7: DAY2 19:00). The semi‐transparent red area indicates the low‐degree range, and the semi‐transparent blue area indicates the high‐degree range. (C–E) Degree maps of TP1, TP5, and TP7. Red patterns indicate high‐degree nodes, while blue patterns indicate low‐degree nodes. The color bars indicate the strength of the nodes’ degree.

Given evidence that critical dynamics can shape large‐scale FC organization, including degree‐dependent coupling features [28, 58, 59], we computed voxel‐wise degree centrality in the FC graph and quantified shifts in the degree distribution (Figure 6A,B). Both the fraction of high‐degree nodes (*F*_(6,150)_ = 19.72, *p* = 6.76e‐17, ηp^2^ = 0.44) and the fraction of low‐degree nodes (*F*_(6,150)_ = 20.21, *p* = 3.05e‐17, ηp^2^ = 0.45) varied significantly during SD. The proportion of high‐degree nodes increased from 6.95% at TP1 to 20.44% at TP5 and then decreased to 14.84% at TP7, with an opposite pattern for low‐degree nodes (TP1: 70.42%; TP5: 51.90%; TP7: 58.75%). Post‐hoc analyses showed that these shifts emerged early, with a significant increase in high‐degree nodes already at TP2 and significant decreases in low‐degree nodes at TP2 and TP3 relative to TP1 (FDR‐corrected *p*s < 0.05; Figure S2A,B). These results indicate that SD induced early (TP2, TP3) and robust shifts in degree distribution, characterized by increased high‐degree and decreased low‐degree nodes relative to TP1.

### 3.4 Criticality Tracks Pressure, Not Lapses

We next tested whether SD–related changes in neuronal avalanche metrics covaried with behavioral measures (sleep pressure, PVT lapse number, and PVT lapse ratio) across time awake. At the whole‐brain level, subjective sleep pressure showed a significant positive similarity with the time courses of mean avalanche size (*t*_22_ = 4.32, *p* = 0.0003, FDR‐corrected *p* = 0.0006, Figure 4C) and branching ratio (*t*_22_ = 4.53, *p* = 0.0002, FDR‐corrected *p* = 0.0002, Figure 4H) during SD. In contrast, the corresponding similarities with psychomotor vigilance lapses were weaker and did not survive FDR correction at the *p* < 0.05 level (PVT lapse number: avalanche size, *t*_19_ = 2.04, *p* = 0.06, FDR‐corrected *p* = 0.09, Figure 4D; branching ratio, *t*_19_ = 1.67, *p* = 0.11, FDR‐corrected *p* = 0.11,, Figure 4I; PVT lapse ratio: avalanche size, *t*_19_ = 2.11, *p* = 0.05, FDR‐corrected *p* = 0.10, Figure 4E; branching ratio, *t*_19_ = 1.75, *p* = 0.10, FDR‐corrected *p* = 0.12, Figure 4J).

To further validate the longitudinal relationships shown in Figure 4, we additionally performed linear mixed‐effects modeling (Table 2). After accounting for the effect of SD duration, both mean avalanche size and branching ratio showed significant associations with subjective sleep pressure (*p* < 0.01). In contrast, neither avalanche metric showed a significant association with PVT lapse number or PVT lapse ratio. These results provide additional support for the observation that avalanche dynamics were more closely related to the longitudinal variation in subjective sleep pressure than to PVT lapse measures.

**TABLE 2:** Linear mixed‐effects models of the longitudinal associations between avalanche metrics and subjective sleep pressure and PVT lapse measures, controlling for sleep deprivation duration.

**Model 1**: SleepPressure_*ij*_ = β_0_ + β_1_AvalanceSize_*ij*_ + β_2_Time_*j*_ + *u_i_* + ε_*ij*_					
	Estimate	SE	*t*‐value	*p*‐value	95% CIs
Intercept	1.6704	0.5441	3.0701	0.0025	[0.5957, 2.7450]
Time	0.1193	0.0128	9.3560	7.8737e‐17	[0.0941, 0.1445]
Avalanche size	0.0817	0.0293	2.7936	0.0059	[0.0239, 0.1395]
**Model 2**: SleepPressure_*ij*_ = β_0_ + β_1_BranchingRatio_*ij*_ + β_2_Time_*j*_ + *u_i_* + ε_*ij*_					
	Estimate	SE	*t*‐value	*p*‐value	95% CIs
Intercept	−0.9916	1.3362	−0.7421	0.4591	[‐3.6306, 1.6475]
Time	0.1199	0.0127	9.4689	3.9562e‐17	[0.0949, 0.1450]
Branching ratio	3.7375	1.2869	2.9043	0.0042	[1.1958, 6.2793]
**Model 3**: PVTlapseNumber_*ij*_ = β_0_ + β_1_AvalanceSize_*ij*_ + β_2_Time_*j*_ + *u_i_* + ε_*ij*_					
	Estimate	SE	*t*‐value	*p*‐value	95% CIs
Intercept	1.1043	1.2016	0.9190	0.3597	[‐1.2718, 3.4803]
Time	0.1806	0.0281	6.4301	1.9688e‐09	[0.1251, 0.2361]
Branching ratio	0.0787	0.0665	1.1834	0.2387	[‐0.0528, 0.2103]
**Model 4**: PVTlapseNumber_*ij*_ = β_0_ + β_1_BranchingRatio_*ij*_ + β_2_Time_*j*_ + *u_i_* + ε_*ij*_					
	Estimate	SE	*t*‐value	*p*‐value	95% CIs
Intercept	−0.2009	2.8735	−0.0699	0.9444	[‐5.8830, 5.4813]
Time	0.1853	0.0277	6.6905	5.2157e‐10	[0.1305, 0.2401]
Branching ratio	2.2984	2.7864	0.8249	0.4109	[‐3.2116, 7.8084]
**Model 5**: PVTlapseRatio_*ij*_ = β_0_ + β_1_AvalanceSize_*ij*_ + β_2_Time_*j*_ + *u_i_* + ε_*ij*_					
	Estimate	SE	*t*‐value	*p*‐value	95% CIs
Intercept	0.0184	0.0230	0.8024	0.4237	[‐0.0270, 0.0639]
Time	0.0034	5.3557e‐04	6.3338	3.1960e‐09	[0.0023, 0.0045]
Branching ratio	0.0014	0.0013	1.0883	0.2784	[‐0.0011, 0.0039]
**Model 6**: PVTlapseRatio_*ij*_ = β_0_ + β_1_BranchingRatio_*ij*_ + β_2_Time_*j*_ + *u_i_* + ε_*ij*_					
	Estimate	SE	*t*‐value	*p*‐value	95% CIs
Intercept	−0.0113	0.0548	−0.2058	0.8373	[‐0.1196, 0.0971]
Time	0.0035	5.2761e‐04	6.5459	1.0937e‐09	[0.0024, 0.0045]
Branching ratio	0.0473	0.0531	0.8906	0.3747	[‐0.0577, 0.1523]

SE = standard error; CI = Confidence interval.

Given the selective coupling between avalanche dynamics and subjective sleep pressure at the whole‐brain level, we next asked whether this relationship exhibited network‐specific organization. To address this question, we repeated the correlation analysis between avalanche dynamics and sleep pressure within each functional network. Sleep pressure showed strong positive similarity with avalanche metrics in the VN (mean avalanche size, *t*_22_ = 6.82, *p* = 7e‐7, FDR‐corrected *p* = 1e‐6; branching ratio, *t*_22_ = 8.03, *p* = 6e‐8, FDR‐corrected *p* = 1e‐7, Figure 5E), and SMN (mean avalanche size, *t*_22_ = 6.76, *p* = 9e‐7, FDR‐corrected *p* = 1e‐6; branching ratio, *t*_22_ = 5.55, *p* = 1e‐5, FDR‐corrected *p* = 1e‐5, Figure 5H) during SD. Across seven functional networks, the LN showed the weakest correspondence with sleep pressure (average avalanche size, *t*_22_ = 2.71, *p* = 0.013, FDR‐corrected *p* = 0.017; branching ratio, *t*_22_ = 2.88, *p* = 0.009, FDR‐corrected *p* = 0.013, Figure 5K). Results for the remaining networks are shown in Figure S1.

Together, these results indicate that SD‐related changes in avalanche dynamics track subjective sleep pressure across networks, with the strongest correspondence in VN and SMN.

### 3.5 Model‐Based Sufficiency of Inhibitory Decay

To test a mechanistic sufficiency account, we asked whether a selective degradation of inhibitory efficacy is sufficient to reproduce the SD‐related drift in propagation regime and its network‐specific heterogeneity. We constructed a recurrent excitatory–inhibitory network model of 1,000 neurons, which was initialized at baseline parameters under which the model operated near a critical regime [35, 52] and expressed spontaneous ∼10 Hz activity (Figure 3A–D). To facilitate comparison with the empirical dynamical shift, we used oscillatory power as a parsimonious model readout of effective recurrent gain and inhibitory control, noting that oscillations and critical dynamics can co‐emerge in recurrent networks with the current prior parameters [60, 61, 62].

To model progressive wakefulness, we introduced a gradual reduction in inhibitory efficacy (Figure 3E), implemented as a linear increase in the firing threshold of inhibitory neurons (*h_I_*) [53, 54], together with a linear decrease in inhibitory synaptic strengths ($W_{IE}^{jk}$, $W_{II}^{jk})\;$[55, 56]. Under this manipulation, gamma‐band power changed robustly across the seven time points (*F*_(6,114)_ = 41.57, *p* = 1.64e‐26, ηp^2^ = 0.69, Figure 3F). Post‐hoc analyses showed that the gamma power increased early in deprivation, with gamma power at TP2 (day 1: 14:00), TP3 (day 1: 19:00), and TP4 (day 1: 22:00) all significantly higher than at TP1 (Day 1: 08:00, FDR‐corrected *ps* < 0.05), followed by a modest numerical decrease later in deprivation (e.g., TP6 to TP7 and TP5 to TP7; not significant) (Figure 3G).

To further examine whether the behavioral relevance of the computational model was consistent with that of the empirical avalanche metrics, we performed additional linear mixed‐effects analyses using model‐derived mean gamma power as the predictor and SD duration as a covariate. Consistent with the avalanche results, mean gamma power significantly predicted subjective sleep pressure, whereas its associations with PVT lapse number and lapse ratio were not significant (Table 3).

**TABLE 3:** Linear mixed‐effects models of the longitudinal associations between gamma power with subjective sleep pressure and PVT lapse measures, controlling for sleep deprivation duration.

**Model 1**: SleepPressure_*ij*_ = β_0_ + β_1_ *Log*(*Gamma* *Power*)_*j*_ + β_2_Time_*j*_ + *u_i_* + ε_*ij*_					
	Estimate	SE	*t*‐value	*p*‐value	95% CIs
Intercept	34.0808	10.4639	3.2570	0.0014	[13.414, 54.748]
Time	0.0556	0.0279	1.9908	0.0482	[0.0004, 0.1108]
Log(GammaPower)	1.7218	0.5742	2.9985	0.0032	[0.5877, 2.8559]
**Model 2**: PVTlapseNumber_*ij*_ = β_0_ + β_1_ *Log*(*Gamma* *Power*)_*j*_ + β_2_Time_*j*_ + *u_i_* + ε_*ij*_					
	Estimate	SE	*t*‐value	*p*‐value	95% CIs
Intercept	8.7163	23.0423	0.3783	0.7058	[‐36.848, 54.281]
Time	0.1764	0.0615	2.8679	0.0048	[0.0548, 0.2980]
Log(GammaPower)	0.3660	1.2644	0.2895	0.7726	[‐2.1342, 2.8663]
**Model 3**: PVTlapseRatio_*ij*_ = β_0_ + β_1_ *Log*(*Gamma* *Power*)_*j*_ + β_2_Time_*j*_ + *u_i_* + ε_*ij*_					
	Estimate	SE	*t*‐value	*p*‐value	95% CIs
Intercept	0.1784	0.4392	0.4063	0.6851	[‐0.6899, 1.0469]
Time	0.0033	0.0012	2.7768	0.0063	[0.0009, 0.0056]
Log(GammaPower)	0.0079	0.0241	0.3267	0.7444	[‐0.0398, 0.0555]

**FIGURE 7:**
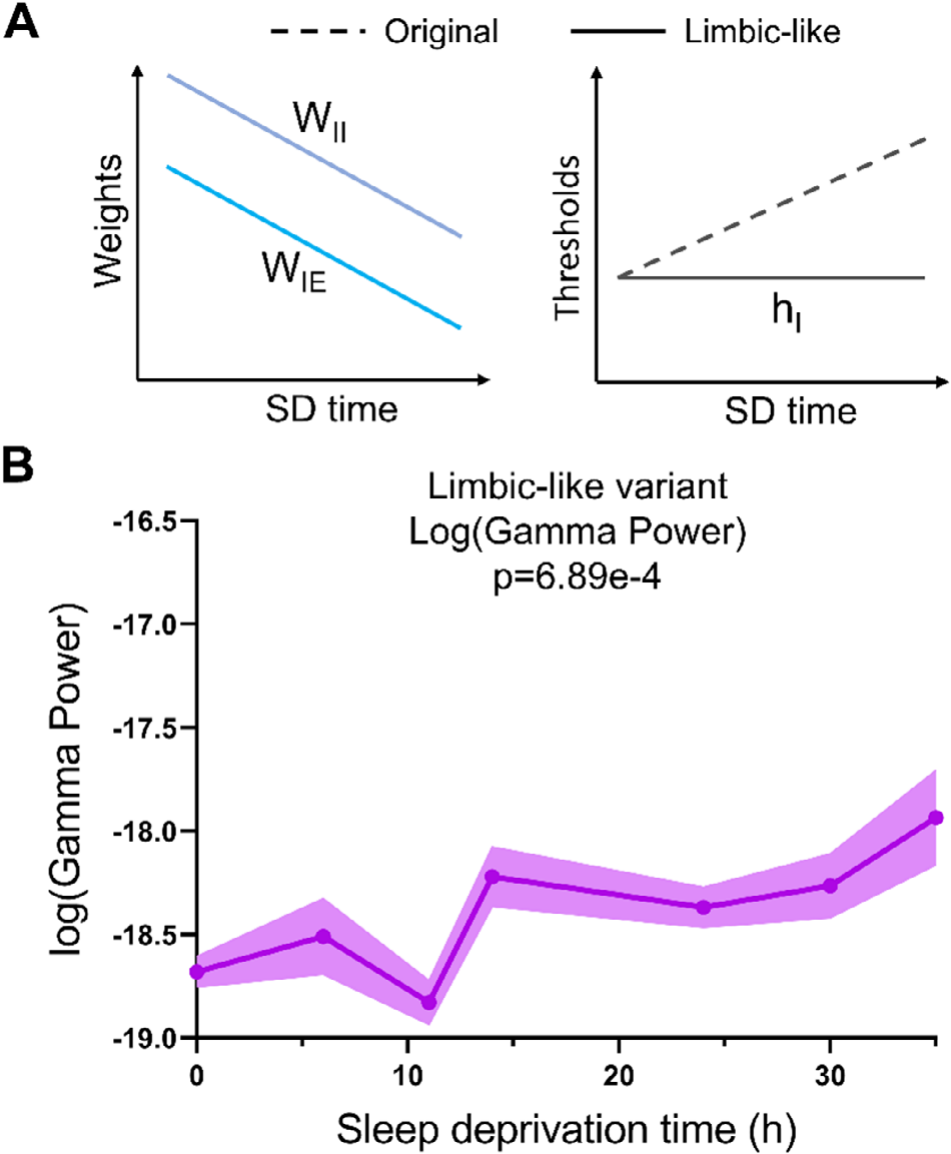
Gamma‐band dynamics of the limbic‐like network model under sleep deprivation without modulation of inhibitory firing threshold. (A) In contrast to the main cortical network model, the firing threshold of inhibitory neurons was fixed at 0 throughout the entire simulation and was not allowed to vary with sleep deprivation duration. (B) Gamma‐band power changes were simulated in the limbic‐like network computational model across different durations of sleep deprivation. Data are shown as mean ± SEM.

We then tested whether the same inhibitory‐decay mechanism can accommodate the network‐level constraint observed empirically, namely, heterogeneous deviations across networks while preserving relative stability in the most resilient systems (i.e., LN). Because inhibitory microcircuits exhibit substantial diversity and area‐specific organization, we allowed inhibitory sensitivity to vary across systems and implemented a limbic‐like variant by setting θ_*h*_ in Equation (13) to 0 [63, 64, 65]. In the limbic‐like variant, the main effect of SD on gamma power was greatly reduced relative to the original model (original model: *F*_(6,114)_ = 41.57, *p* = 1.64e‐26, ηp^2^ = 0.69; limbic‐like variant: *F*_(6,114)_ = 4.24, *p* = 6.89e‐4, ηp^2^ = 0.18; Figure 7), indicating a flatter time course of gamma power across deprivation, consistent with the relative stability of avalanche metrics observed in the limbic network.

Together, these modeling results indicate that progressive inhibitory decay yields SD‐like gamma‐power changes, whereas reduced inhibitory sensitivity produces a flatter deprivation trajectory.

### 3.6 Comparison Models for Testing the Specificity of the Inhibitory‐Decay Account

To assess whether the empirical temporal profile could also arise from alternative Excitation/Inhibition (E/I) perturbations, we compared the IDCNM with an excitatory‐enhancement model and a mixed E/I model. Across simulations, all three manipulations shifted the model away from its baseline operating regime. However, within the present framework, the inhibitory‐decay model better captured the empirical pattern. Besides reproducing the overall direction of change, it also showed a later‐stage plateau and partial rebound. By contrast, the excitatory‐enhancement and mixed models yielded a more monotonic increase in the model proxy (Supporting Text and Figure S9). These results suggest that selective inhibitory decay provides a more plausible account of the observed phenomenology than the alternative perturbation classes tested here. An additional reversed‐weight control analysis showed that swapping the initial E/I weight structure led to immediate saturation and degenerate gamma estimates, indicating that the model depends critically on the original E/I assignment (Supporting Text and Figure S10).

## 4 Discussion

To our knowledge, our study provides one of the first time‐resolved characterizations of the trajectory of human brain criticality during prolonged SD. Using neuronal avalanche analysis of rs‐fMRI data sampled at seven time points over 36‐h of continuous wakefulness, we observed a progressive drift in propagation dynamics from near‐critical toward supercritical regimes at the whole‐brain level. This drift was not spatially uniform. Visual and sensorimotor systems showed the largest deviations, whereas the limbic network remained comparatively stable. Critically, criticality changes tracked accumulated subjective sleep pressure but were largely dissociated from psychomotor vigilance lapses. Finally, a minimal recurrent E–I network model showed that progressive inhibitory decay was sufficient to reproduce an SD‐like dynamical shift and a limbic‐like resilience pattern, providing a constrained mechanistic interpretation linking sleep pressure to large‐scale propagation stability.

Within a criticality framework, these findings can be interpreted as follows. Across deprivation, both branching ratio and mean avalanche size increased, consistent with an amplification of activity propagation and a shift away from near‐critical dynamics. In a branching‐process view, σ > 1 reflects amplified propagation, compatible with weakened control over effective recurrent gain. Such a drift is consistent with a progressive disruption of excitation–inhibition regulation that normally stabilizes large‐scale dynamics [66, 67]. Interestingly, avalanche metrics did not increase indefinitely and showed no further increase over the final sampling interval, suggesting that SD pushes the system toward an altered operating regime that may approach a quasi‐steady state over the observed window. However, longer deprivation will be required to determine whether dynamics continue to drift beyond 36 h or vary across circadian phase [68, 69]. Similar deviations from near‐critical dynamics have been reported in clinical states such as epilepsy and depression [29, 30], supporting the broader view that distance from near‐criticality can serve as a dimensional marker of brain functional state.

At the empirical level, sleep pressure increased beginning on Day 1, whereas prominent vigilance impairment emerged later. Against this background, whole‐brain avalanche metrics covaried more closely with sleep pressure than with PVT lapses. In particular, mean avalanche size increased early on Day 1, paralleling the early rise in subjective sleep pressure. This temporal dissociation suggests that criticality metrics are sensitive to the gradual homeostatic component of SD rather than being driven primarily by moment‐to‐moment performance failures [70]. This framing is consistent with sleep‐regulation theory in which a sleep‐wake‐dependent homeostatic process interacts with a circadian process that can partially mask or modulate behavioral impairments across the day [68, 71]. Within this perspective, avalanche‐based criticality may provide a dynamical readout of sleep homeostasis that complements conventional behavioral assays [72, 73].

At the functional‐network level, SD induced heterogeneous deviations in propagation regime. The VN and SMN showed the largest increases in branching ratio and mean avalanche size, whereas the LN remained near criticality. This pattern is consistent with evidence that sensory and sensorimotor systems are particularly sensitive to sleep loss and altered cortical excitability, which may reflect compensatory engagement to support performance under challenge [74, 75, 76, 77, 78]. In contrast, the relative stability of LN dynamics suggests that SD does not impose a uniform global shift in propagation gain, but instead reshapes propagation stability in a network‐dependent manner. A plausible interpretation is that limbic circuits may be comparatively resilient to the specific control‐parameter changes induced by SD, potentially due to differences in inhibitory microcircuit organization and neuromodulatory regulation [65, 79, 80, 81, 82]. This interpretation remains tentative given the macroscopic nature of fMRI‐derived measures, and it motivates targeted tests of region‐specific inhibitory and neuromodulatory contributions under SD. Importantly, these network‐specific effects impose a strong constraint on mechanistic accounts, such that any candidate circuit‐level manipulation should capture both the global drift and the relative stability of limbic dynamics. In parallel with dynamical changes, SD was accompanied by a robust reorganization of functional network topology. Specifically, the fraction of high‐degree nodes increased while the fraction of low‐degree nodes decreased, indicating a shift in the degree distribution toward greater hubness. Such redistribution is consistent with increased global coupling and reduced heterogeneity of network interactions, and may accompany altered propagation stability [83, 84]. Notably, these degree‐distribution shifts emerged early in deprivation, matching the early deviation in avalanche metrics. While the present data are correlational, the co‐evolution of topology and propagation regime suggests that SD involves coordinated changes in both dynamic and network‐topological metrics of large‐scale brain organization.

To probe a circuit‐level mechanism, we performed a mechanistic sufficiency analysis in a recurrent excitatory‐inhibitory cortical network model. Selectively degrading inhibitory efficacy, implemented as a gradual elevation of inhibitory firing thresholds together with a proportional reduction in inhibitory synaptic strength [53, 54, 55, 56], was sufficient to reproduce SD‐like changes in a simple dynamical proxy, i.e., gamma‐band power, consistent with a progressive increase in effective recurrent gain. Within the present model, this selective inhibitory decay was sufficient to generate the observed direction of change without introducing additional source‐input or alterations to network topology. Importantly, the model also reproduced a key network‐level constraint, in that reducing inhibitory sensitivity in a limbic‐like variant substantially weakened the deprivation effect on gamma power, consistent with the relative stability of limbic avalanche dynamics observed empirically. This pattern is consistent with a constrained mechanistic account whereby accumulated sleep pressure corresponds to a progressive loss of inhibitory control that destabilizes large‐scale propagation and drives the system toward supercritical dynamics. Here, gamma‐band power serves as an interpretable model output indexing effective gain and inhibitory control, rather than as a quantitative fit to the empirical time courses of avalanche metrics or sleep pressure.

This mechanistic account is consistent with human neurophysiological evidence that SD alters cortical excitability and inhibition–facilitation regulation. Using paired‐pulse Transcranial Magnetic Stimulation (TMS) over primary motor cortex, Civardi et al. [85] found that SD significantly reduced both intracortical inhibition and intracortical facilitation [85], indicating a change in inhibition–facilitation balance. More broadly, Salehinejad et al. [86] reported that SD upscales cortical excitability, attributing this effect to enhanced glutamate‐related cortical facilitation and reduced (and potentially reversed) GABAergic cortical inhibition [86]. In the same study, Salehinejad et al. also found that SD altered the inducibility of transcranial direct‐current stimulation driven (tDCS‐driven) long‐term potentiation (LTP) or long‐term depression like (LTD) plasticity, including abolished anodal LTP‐like effects and a conversion of cathodal LTD‐like effects toward LTP‐like plasticity. Importantly, reported effects can vary across studies because excitability and inhibition are indexed by multiple stimulation protocols and outcome metrics that probe partially distinct physiological processes, ranging from corticospinal thresholds and input–output (I–O) curves to paired‐pulse measures such as short‐interval intracortical inhibition (SICI), intracortical facilitation (ICF), I‐wave facilitation, and Short‐Latency Afferent Inhibition (SAI) [86, 87, 88, 89]. Moreover, these estimates are sensitive to specific parameter choices, including stimulation intensity, interstimulus interval, and state dependence, which can further contribute to heterogeneity across studies [90, 91]. Therefore, future work will benefit from multimodal validation and from explicitly specifying how excitability and inhibition are operationalized when comparing results across methods and protocols.

The present model is informative not only because it shows that selective inhibitory weakening can reproduce an SD‐like shift in a dynamical proxy, but also because it helps refine the space of plausible circuit‐level hypotheses. In principle, a drift away from near‐critical dynamics during SD could arise from multiple classes of perturbation, including enhanced excitation, weakened inhibition, or combined E/I alterations. However, these perturbation classes are not dynamically equivalent. Within the present framework, selective inhibitory weakening better captures the empirical combination of whole‐brain drift, a later‐stage plateau, and partial rebound. By contrast, a purely excitatory‐enhancement account would be expected to produce a broader and more monotonic amplification of network activity, whereas mixed mechanisms may also contribute but would require additional assumptions to explain the observed temporal pattern.

This more differentiated view also generates testable predictions. First, if inhibitory weakening contributes substantially to the observed SD‐related state shift, then interventions or perturbations expected to enhance inhibitory efficacy should bias empirical avalanche metrics toward a less supercritical regime, potentially with larger effects in networks showing the greatest SD‐related deviations (e.g., VN/SMN). This prediction is model‐derived and remains to be tested directly. Second, avalanche‐based criticality metrics and oscillatory measures may reflect partly shared network processes related to effective gain and inhibitory stabilization [61, 92, 93]. If so, SD should produce partly matching changes across modalities, for example, shifts in EEG or Magnetoencephalography (MEG) propagation‐related measures together with changes in independent measures of cortical excitability and inhibition, without requiring a one‐to‐one correspondence across modalities or brain regions [6]. More generally, the model suggests that future perturbation studies should not only ask whether SD alters E/I balance, but also whether distinct forms of E/I disruption produce distinguishable signatures in the temporal trajectory. It should be noted that while the empirical data and criticality‐based interpretation are directly supported by observations, the circuit‐level account represents a model‐based and non‐unique mechanistic explanation.

How might sleep pressure map onto a control parameter that modulates the propagation regime? One candidate bridge is homeostatic synaptic regulation, which proposes that prolonged wakefulness can be accompanied by net synaptic potentiation and increased effective coupling, whereas sleep may restore synaptic balance through downscaling [94, 95]. In this context, the drift toward supercritical propagation observed here may reflect a macroscale signature of wake‐dependent increases in effective gain and/or reduced inhibitory stabilization accumulating with sleep pressure. Although the present study did not directly quantify synaptic strength or slow‐wave activity, integrating avalanche‐based measures with electrophysiological markers of homeostasis in future work will be important for determining how synaptic and inhibitory processes jointly shape large‐scale propagation stability.

This study has several limitations. First, although the protocol involved intensive within‐subject sampling, the sample size remains modest, and replication in larger and more diverse cohorts will be important for generalization. Second, our cohort consisted of healthy young adults. Future work should test whether similar dynamical trajectories and network‐specific resilience patterns generalize to old cohorts and to clinical populations with sleep disorders. Third, BOLD‐derived avalanches provide an operational estimate of propagation regime from rs‐fMRI but remain an indirect measure of neuronal dynamics, and avalanche statistics can be influenced by temporal resolution, preprocessing choices, and thresholding procedures. Converging multimodal data (e.g., EEG/MEG), therefore, will be valuable for linking criticality shifts more directly to electrophysiological mechanisms [27, 28, 72]. Fourth, brief microsleeps or transient N1 episodes during scanning cannot be fully excluded in the present study, though continuous video monitoring and eyes‐open fixation were used. Therefore, future studies should combine simultaneous EEG/EOG and/or pupillometry with fast‐fMRI to obtain objective arousal indices and verify our present findings [96, 97, 98]. Fifth, because the 01:00 and 04:00 sessions likely sampled the biological night, when homeostatic sleep pressure and circadian sleep promotion jointly exert strong effects, excluding them from the primary analysis limited our ability to characterize the full overnight profile of deprivation‐related brain dynamics and to dissociate homeostatic from circadian influences. Sixth, a further limitation is that avalanche events were defined using fixed group‐level thresholds rather than participant‐specific thresholds. Although this choice improved the stability and comparability of group‐level estimation, it may have reduced sensitivity to inter‐individual differences in optimal thresholding and, therefore, limited the precision of individual trajectory characterization. Future studies with longer fMRI acquisitions and denser avalanche sampling will be better positioned to test participant‐specific thresholding strategies. Finally, our computational model is minimal by design and uses gamma‐band power as an interpretable dynamical proxy. The present computational model should be interpreted as a conceptual demonstration of mechanistic plausibility and a mechanistic sufficiency framework, rather than as a direct generator of the empirical avalanche observables. Its ability to reproduce key empirical signatures under constrained assumptions supports inhibitory impairment as a plausible candidate mechanism under constrained modelling assumption, but does not exclude additional contributions (e.g., neuromodulation, structural constraints, or circadian gating) that may shape network‐specific dynamics under SD.

In summary, SD induced a systematic drift of human brain dynamics away from near‐criticality toward a supercritical regime, accompanied by network‐level reorganization and pronounced heterogeneity across functional systems. By integrating longitudinal rs‐fMRI avalanche analysis with a simple circuit‐interpretable model, we provide evidence consistent with progressive inhibitory impairment as a mechanistic candidate linking accumulated sleep pressure to large‐scale propagation instability, while accounting for relative limbic resilience. Together, these findings highlight criticality metrics as sensitive dynamical markers of SD‐related brain‐state vulnerability and underscore the importance of inhibitory regulation in maintaining stable cortical dynamics under physiological challenge.

## Funding

This work was supported by the Brain Science and Brain‐like Intelligence Technology – National Science and Technology Major Project (2021ZD0203803), National Natural Science Foundation of China (32300869, 32200840, 32271108), National Human Genetic Resources Sharing Service Platform (2005DKA21300), and Fundamental Research Funds for the Central Universities.

## Conflicts of Interest

The authors declare no conflicts of interest.

## Supporting information




**Supporting File**: advs75698‐sup‐0001‐SuppMat.docx.

## Data Availability

The data that support the findings of this study are available on request from the corresponding author. The data are not publicly available due to privacy or ethical restrictions.

## References

[1] J. S. Durmer and D. F. Dinges , “Neurocognitive Consequences of Sleep Deprivation,” Seminars in Neurology 25, no. 1 (2005): 117–129. 15798944 10.1055/s-2005-867080

[2] W. D. S. Killgore , “Effects of Sleep Deprivation on Cognition,” Progress in Brain Research 185 (2010): 105–129. 21075236 10.1016/B978-0-444-53702-7.00007-5

[3] A. J. Krause , E. B. Simon , B. A. Mander , et al., “The Sleep‐Deprived Human Brain,” Nature Reviews Neuroscience 18, no. 7 (2017): 404–418. 28515433 10.1038/nrn.2017.55 PMC6143346

[4] C. J. Lowe , A. Safati , and P. A. Hall , “The Neurocognitive Consequences of Sleep Restriction: A Meta‐Analytic Review,” Neuroscience & Biobehavioral Reviews 80 (2017): 586–604. 28757454 10.1016/j.neubiorev.2017.07.010

[5] Z. Yang , S. D. Williams , E. Beldzik , S. Anakwe , E. Schimmelpfennig , and L. D. Lewis , “Attentional Failures after Sleep Deprivation Are Locked to Joint Neurovascular, Pupil and Cerebrospinal Fluid Flow Dynamics,” Nature Neuroscience 28 (2025): 2526–2536. 41162627 10.1038/s41593-025-02098-8 PMC12672380

[6] J. Q. M. Ly , G. Gaggioni , S. L. Chellappa , et al., “Circadian Regulation of human Cortical Excitability,” Nature Communications 7, no. 1 (2016): 11828. 10.1038/ncomms11828 PMC4931032 27339884

[7] M. Kuhn , F. Mainberger , B. Feige , et al., “State‐Dependent Partial Occlusion of Cortical LTP‐Like Plasticity in Major Depression,” Neuropsychopharmacology 41, no. 6 (2016): 1521–1529. 26442602 10.1038/npp.2015.310 PMC4832013

[8] J. T. Weiss , M. Z. Blundell , P. Singh , and J. M. Donlea , “Sleep Deprivation Drives Brain‐Wide Changes in Cholinergic Presynapse Abundance in Drosophila Melanogaster,” Proceedings of the National Academy of Sciences 121, no. 13 (2024): 2312664121. 10.1073/pnas.2312664121 PMC10990117 38498719

[9] F. Barkhof , S. Haller , and S. A. R. B. Rombouts , “Resting‐State Functional MR Imaging: A New Window to the Brain,” Radiology 272, no. 1 (2014): 29–49. 24956047 10.1148/radiol.14132388

[10] M. H. Lee , C. D. Smyser , and J. S. Shimony , “Resting‐State fMRI: A Review of Methods and Clinical Applications,” American Journal of Neuroradiology 34, no. 10 (2013): 1866–1872. 22936095 10.3174/ajnr.A3263 PMC4035703

[11] W.‐H. Chen , J. Chen , X. Lin , et al., “Dissociable Effects of Sleep Deprivation on Functional Connectivity in the Dorsal and Ventral Default Mode Networks,” Sleep Medicine 50 (2018): 137–144. 30055480 10.1016/j.sleep.2018.05.040

[12] S. Khalsa , S. D. Mayhew , I. Przezdzik , et al., “Variability in Cumulative Habitual Sleep Duration Predicts Waking Functional Connectivity,” Sleep 39, no. 1 (2016): 87–95. 26414900 10.5665/sleep.5324 PMC4678343

[13] Y. Lei , Y. Shao , L. Wang , et al., “Large‐Scale Brain Network Coupling Predicts Total Sleep Deprivation Effects on Cognitive Capacity,” PLoS ONE 10, no. 7 (2015): 0133959. 10.1371/journal.pone.0133959 PMC4517902 26218521

[14] J. Lim , J. C. Tan , S. Parimal , D. F. Dinges , and M. W. L. Chee , “Sleep Deprivation Impairs Object‐Selective Attention: A View from the Ventral Visual Cortex,” PLoS ONE 5, no. 2 (2010): 9087. 10.1371/journal.pone.0009087 PMC2816724 20140099

[15] C. W. Wu , P.‐Y. Liu , P.‐J. Tsai , et al., “Variations in Connectivity in the Sensorimotor and Default‐Mode Networks during the First Nocturnal Sleep Cycle,” Brain Connectivity 2, no. 4 (2012): 177–190. 22817652 10.1089/brain.2012.0075

[16] X. Zhang , X. Wang , J. Bao , and Y. Liu , “Modular Network Analysis of Abnormal Functional Connectivity and Disrupted Brain Topology Caused by Sleep Deprivation and Its Association with Cognitive Function,” Sleep Medicine 133 (2025): 106514. 40578294 10.1016/j.sleep.2025.106514

[17] K. J. Friston , “Functional and Effective Connectivity: A Review,” Brain Connectivity 1, no. 1 (2011): 13–36. 22432952 10.1089/brain.2011.0008

[18] M. N. Hallquist and F. G. Hillary , “Graph Theory Approaches to Functional Network Organization in Brain Disorders: A Critique for a Brave New Small‐World,” Network Neuroscience 3, no. 1 (2018): 1–26. 30793071 10.1162/netn_a_00054 PMC6326733

[19] S. M. Smith , D. Vidaurre , C. F. Beckmann , et al., “Functional Connectomics from Resting‐State fMRI,” Trends in Cognitive Sciences 17, no. 12 (2013): 666–682. 24238796 10.1016/j.tics.2013.09.016 PMC4004765

[20] R. M. Hutchison , T. Womelsdorf , E. A. Allen , et al., “Dynamic Functional Connectivity: Promise, Issues, and Interpretations,” Neuroimage 80 (2013): 360–378. 23707587 10.1016/j.neuroimage.2013.05.079 PMC3807588

[21] A.‐E. Avramiea , A. Masood , H. D. Mansvelder , and K. Linkenkaer‐Hansen , “Long‐Range Amplitude Coupling Is Optimized for Brain Networks That Function at Criticality,” The Journal of Neuroscience 42, no. 11 (2022): 2221–2233. 35082120 10.1523/JNEUROSCI.1095-21.2022 PMC8936577

[22] L. Cocchi , L. L. Gollo , A. Zalesky , and M. Breakspear , “Criticality in the Brain: A Synthesis of Neurobiology, Models and Cognition,” Progress in Neurobiology 158 (2017): 132–152. 28734836 10.1016/j.pneurobio.2017.07.002

[23] J. O'Byrne and K. Jerbi , “How Critical Is Brain Criticality?,” Trends in Neurosciences 45, no. 11 (2022): 820–837. 36096888 10.1016/j.tins.2022.08.007

[24] J. M. Beggs and N. Timme , “Being Critical of Criticality in the Brain,” Frontiers in Physiology 3 (2012): 163. 22701101 10.3389/fphys.2012.00163 PMC3369250

[25] J. M. Beggs , “The Criticality Hypothesis: How Local Cortical Networks Might Optimize Information Processing,” Philosophical Transactions of the Royal Society A: Mathematical, Physical and Engineering Sciences 366 (1864): 329–343. 10.1098/rsta.2007.2092 17673410

[26] W. L. Shew , H. Yang , S. Yu , R. Roy , and D. Plenz , “Information Capacity and Transmission Are Maximized in Balanced Cortical Networks with Neuronal Avalanches,” The Journal of Neuroscience 31, no. 1 (2011): 55–63. 21209189 10.1523/JNEUROSCI.4637-10.2011 PMC3082868

[27] O. Shriki , J. Alstott , F. Carver , et al., “Neuronal Avalanches in the Resting MEG of the human Brain,” The Journal of Neuroscience 33, no. 16 (2013): 7079–7090. 23595765 10.1523/JNEUROSCI.4286-12.2013 PMC3665287

[28] E. Tagliazucchi , P. Balenzuela , D. Fraiman , and D. R. Chialvo , “Criticality in Large‐Scale Brain fMRI Dynamics Unveiled by a Novel Point Process Analysis,” Frontiers in Physiology 3 (2012): 15. 22347863 10.3389/fphys.2012.00015 PMC3274757

[29] C. Meisel , A. Storch , S. Hallmeyer‐Elgner , E. Bullmore , and T. Gross , “Failure of Adaptive Self‐Organized Criticality during Epileptic Seizure Attacks,” PLoS Computational Biology 8, no. 1 (2012): 1002312. 10.1371/journal.pcbi.1002312 PMC3252275 22241971

[30] Y. Xin , T. Bai , T. Zhang , et al., “Electroconvulsive Therapy Modulates Critical Brain Dynamics in Major Depressive Disorder Patients,” Brain Stimulation 15, no. 1 (2022): 214–225, 10.1016/j.brs.2021.12.008. 34954084

[31] X. Liu , X. Fei , and J. Liu , “The Cognitive Critical Brain: Modulation of Criticality in Perception‐Related Cortical Regions,” Neuroimage 305 (2025): 120964. 39643023 10.1016/j.neuroimage.2024.120964

[32] M. Basner , D. Mollicone , and D. F. Dinges , “Validity and Sensitivity of a Brief Psychomotor Vigilance Test (PVT‐B) to Total and Partial Sleep Deprivation,” Acta Astronautica 69, no. 11‐12 (2011): 949–959, 10.1016/j.actaastro.2011.07.015. 22025811 PMC3197786

[33] D. F. Dinges , F. Pack , K. Williams , et al., “Cumulative Sleepiness, Mood Disturbance, and Psychomotor Vigilance Performance Decrements during a Week of Sleep Restricted to 4–5 Hours per Night,” Sleep 20, no. 4 (1997): 267–277. 9231952

[34] J. Lim and D. F. Dinges , “Sleep Deprivation and Vigilant Attention,” Annals of the New York Academy of Sciences 1129 (2008): 305–322, 10.1196/annals.1417.002. 18591490

[35] B. Yang , H. Zhang , T. Jiang , and S. Yu , “Natural Brain State Change with E/I Balance Shifting Toward Inhibition is Associated with Vigilance Impairment,” Iscience 26, no. 10 (2023): 107963. 37822500 10.1016/j.isci.2023.107963 PMC10562778

[36] J. S. Isaacson and M. Scanziani , “How Inhibition Shapes Cortical Activity,” Neuron 72, no. 2 (2011): 231–243. 22017986 10.1016/j.neuron.2011.09.027 PMC3236361

[37] T. P. Vogels , H. Sprekeler , F. Zenke , C. Clopath , and W. Gerstner , “Inhibitory Plasticity Balances Excitation and Inhibition in Sensory Pathways and Memory Networks,” Science 334, no. 6062 (2011): 1569–1573. 22075724 10.1126/science.1211095

[38] D. Zhang , R. Wang , L. Zhou , K. Zhou , Z. Zuo , and G. Sun , “Biphasic Adaptation of gBOLD‐CSF Coupling during Sleep Deprivation Reflects Compensatory Enhancement and Temporal Disruption in Glymphatic Function,” Neuroimage 328 (2026): 121769. 41638414 10.1016/j.neuroimage.2026.121769

[39] C.‐A. Park , C.‐K. Kang , Y.‐D. Son , et al., “The Effects of Caffeine Ingestion on Cortical Areas: Functional Imaging Study,” Magnetic Resonance Imaging 32, no. 4 (2014): 366–371. 24512799 10.1016/j.mri.2013.12.018

[40] A. L. Rack‐Gomer , J. Liau , and T. T. Liu , “Caffeine Reduces Resting‐State BOLD Functional Connectivity in the Motor Cortex,” Neuroimage 46, no. 1 (2009): 56–63. 19457356 10.1016/j.neuroimage.2009.02.001 PMC2686062

[41] A. M. Tucker , P. Whitney , G. Belenky , J. M. Hinson , and H. P. Van Dongen , “Effects of Sleep Deprivation on Dissociated Components of Executive Functioning,” Sleep 33, no. 1 (2010): 47–57. 20120620 10.1093/sleep/33.1.47 PMC2802247

[42] P. Whitney , J. M. Hinson , B. C. Satterfield , D. A. Grant , K. A. Honn , and H. P. Van Dongen , “Sleep Deprivation Diminishes Attentional Control Effectiveness and Impairs Flexible Adaptation to Changing Conditions,” Scientific Reports 7, no. 1 (2017): 16020. 29167485 10.1038/s41598-017-16165-z PMC5700060

[43] J. Thomann , C. R. Baumann , H. P. Landolt , and E. Werth , “Psychomotor Vigilance Task Demonstrates Impaired Vigilance in Disorders with Excessive Daytime Sleepiness,” Journal of Clinical Sleep Medicine 10, no. 9 (2014): 1019–1024, 10.5664/jcsm.4042. 25142762 PMC4153121

[44] S. R. Gohel and B. B. Biswal , “Functional Integration between Brain Regions at Rest Occurs in Multiple‐Frequency Bands,” Brain Connectivity 5, no. 1 (2015): 23–34. 24702246 10.1089/brain.2013.0210 PMC4313418

[45] C. Trapp , K. Vakamudi , and S. Posse , “On the Detection of High Frequency Correlations in Resting State fMRI,” Neuroimage 164 (2018): 202–213. 28163143 10.1016/j.neuroimage.2017.01.059 PMC5540810

[46] A. Klaus , S. Yu , and D. Plenz , “Statistical Analyses Support Power Law Distributions Found in Neuronal Avalanches,” PLoS ONE 6, no. 5 (2011): 19779. 10.1371/journal.pone.0019779 PMC3102672 21720544

[47] B. T. T. Yeo , F. M. Krienen , J. Sepulcre , et al., “The Organization of the Human Cerebral Cortex Estimated by Intrinsic Functional Connectivity,” Journal of Neurophysiology 106 (2011): 1125–1165. 21653723 10.1152/jn.00338.2011 PMC3174820

[48] A. Helmstetter and D. Sornette , “Subcritical and Supercritical Regimes in Epidemic Models of Earthquake Aftershocks,” Journal of Geophysical Research: Solid Earth 107, no. B10 (2002): ESE–10.

[49] M. Rubinov and O. Sporns , “Complex Network Measures of Brain Connectivity: Uses and Interpretations,” Neuroimage 52, no. 3 (2010): 1059–1069. 19819337 10.1016/j.neuroimage.2009.10.003

[50] X.‐N. Zuo , R. Ehmke , M. Mennes , et al., “Network Centrality in the Human Functional Connectome,” Cerebral Cortex 22, no. 8 (2012): 1862–1875. 21968567 10.1093/cercor/bhr269

[51] P. Qin , X. Wu , C. Wu , et al., “Higher‐Order Sensorimotor Circuit of the Brain's Global Network Supports Human Consciousness,” Neuroimage 231 (2021): 117850. 33582277 10.1016/j.neuroimage.2021.117850 PMC9583596

[52] C. S. Herrmann , M. M. Murray , S. Ionta , A. Hutt , and J. Lefebvre , “Shaping Intrinsic Neural Oscillations with Periodic Stimulation,” The Journal of Neuroscience 36, no. 19 (2016): 5328–5337. 27170129 10.1523/JNEUROSCI.0236-16.2016 PMC6601804

[53] P. T. Morgan , E. F. Pace‐Schott , G. F. Mason , et al., “Cortical GABA Levels in Primary Insomnia,” Sleep 35, no. 6 (2012): 807–814, 10.5665/sleep.1880. 22654200 PMC3353043

[54] Y. Zhang , Y. Shi , Y. Zhang , J. Jiao , and X. Tang , “Cortical Excitability on Sleep Deprivation Measured by Transcranial Magnetic Stimulation: A Systematic Review and Meta‐Analysis,” Brain Research Bulletin 221 (2025): 111190. 39756660 10.1016/j.brainresbull.2025.111190

[55] P. Kreuzer , B. Langguth , R. Popp , et al., “Reduced Intra‐Cortical Inhibition after Sleep Deprivation: A Transcranial Magnetic Stimulation Study,” Neuroscience Letters 493, no. 3 (2011): 63–66. 21352891 10.1016/j.neulet.2011.02.044

[56] P. Manganotti , A. Palermo , S. Patuzzo , G. Zanette , and A. Fiaschi , “Decrease in Motor Cortical Excitability in Human Subjects after Sleep Deprivation,” Neuroscience Letters 304, no. 3 (2001): 153–156. 11343825 10.1016/s0304-3940(01)01783-9

[57] G. Turrigiano , “Homeostatic Synaptic Plasticity: Local and Global Mechanisms for Stabilizing Neuronal Function,” in Cold Spring Harbor perspectives in biology (Cold Spring Harbor Lab Press, 2012), a005736. 10.1101/cshperspect.a005736 PMC3249629 22086977

[58] A. Haimovici , E. Tagliazucchi , P. Balenzuela , and D. R. Chialvo , “Brain Organization into Resting State Networks Emerges at Criticality<? Format?>on a Model of the Human Connectome,” Physical Review Letters 110, no. 17 (2013): 178101. 23679783 10.1103/PhysRevLett.110.178101

[59] H. Lee , D. Golkowski , D. Jordan , et al., “Relationship of Critical Dynamics, Functional Connectivity, and States of Consciousness in Large‐Scale human Brain Networks,” Neuroimage 188 (2019): 228–238. 30529630 10.1016/j.neuroimage.2018.12.011

[60] G. Buzsáki and X.‐J. Wang , “Mechanisms of Gamma Oscillations,” Annual Review of Neuroscience 35, no. 1 (2012): 203–225. 10.1146/annurev-neuro-062111-150444 PMC4049541 22443509

[61] S.‐S. Poil , R. Hardstone , H. D. Mansvelder , and K. Linkenkaer‐Hansen , “Critical‐State Dynamics of Avalanches and Oscillations Jointly Emerge from Balanced Excitation/Inhibition in Neuronal Networks,” Journal of Neuroscience 32, no. 29 (2012): 9817–9823. 22815496 10.1523/JNEUROSCI.5990-11.2012 PMC3553543

[62] X.‐J. Wang and G. Buzsáki , “Gamma Oscillation by Synaptic Inhibition in a Hippocampal Interneuronal Network Model,” The Journal of Neuroscience 16, no. 20 (1996): 6402–6413. 8815919 10.1523/JNEUROSCI.16-20-06402.1996 PMC6578902

[63] L. Lim , D. Mi , A. Llorca , and O. Marín , “Development and Functional Diversification of Cortical Interneurons,” Neuron 100, no. 2 (2018): 294–313. 30359598 10.1016/j.neuron.2018.10.009 PMC6290988

[64] B. Tasic , Z. Yao , L. T. Graybuck , et al., “Shared and Distinct Transcriptomic Cell Types across Neocortical Areas,” Nature 563, no. 7729 (2018): 72–78. 30382198 10.1038/s41586-018-0654-5 PMC6456269

[65] R. Tremblay , S. Lee , and B. Rudy , “GABAergic Interneurons in the Neocortex: from Cellular Properties to Circuits,” Neuron 91, no. 2 (2016): 260–292. 27477017 10.1016/j.neuron.2016.06.033 PMC4980915

[66] D. R. Chialvo , “Critical Brain Networks,” Physica A: Statistical Mechanics and its Applications 340, no. 4 (2004): 756–765.

[67] A. Mora‐Sánchez , G. Dreyfus , and F.‐B. Vialatte , “Scale‐Free Behaviour and Metastable Brain‐State Switching Driven by Human Cognition, an Empirical Approach,” Cognitive Neurodynamics 13 (2019): 437–452. 31565089 10.1007/s11571-019-09533-0 PMC6746897

[68] A. A. Borbély , S. Daan , A. Wirz‐Justice , and T. Deboer , “The Two‐Process Model of Sleep Regulation: A Reappraisal,” Journal of Sleep Research 25, no. 2 (2016): 131–143. 26762182 10.1111/jsr.12371

[69] N. Goel , M. Basner , H. Rao , and D. F. Dinges , “Circadian Rhythms, Sleep Deprivation, and Human Performance,” Progress in Molecular Biology and Translational Science 119 (2013): 155–190. 23899598 10.1016/B978-0-12-396971-2.00007-5 PMC3963479

[70] T. Porkka‐Heiskanen , “Sleep Homeostasis,” Current Opinion in Neurobiology 23, no. 5 (2013): 799–805. 23510741 10.1016/j.conb.2013.02.010

[71] A. A. Borbély , “A Two Process Model of Sleep Regulation,” Human Neurobiology 1, no. 3 (1982): 195–204. 7185792

[72] J. M. Beggs and D. Plenz , “Neuronal Avalanches in Neocortical Circuits,” The Journal of Neuroscience 23, no. 35 (2003): 11167–11177. 14657176 10.1523/JNEUROSCI.23-35-11167.2003 PMC6741045

[73] F. Lombardi , M. Gómez‐Extremera , P. Bernaola‐Galván , et al., “Critical Dynamics and Coupling in Bursts of Cortical Rhythms Indicate Non‐Homeostatic Mechanism for Sleep‐Stage Transitions and Dual Role of VLPO Neurons in both Sleep and Wake,” The Journal of Neuroscience 40, no. 1 (2020): 171–190. 31694962 10.1523/JNEUROSCI.1278-19.2019 PMC6939478

[74] C.‐H. Chia , X.‐W. Tang , Y. Cao , et al., “Cortical Excitability Signatures for the Degree of Sleepiness in Human,” elife 10 (2021): 65099. 10.7554/eLife.65099 PMC8373378 34313218

[75] S. P. A. Drummond , M. J. Meloy , M. A. Yanagi , H. J. Orff , and G. G. Brown , “Compensatory Recruitment after Sleep Deprivation and the Relationship with Performance,” Psychiatry Research: Neuroimaging 140, no. 3 (2005): 211–223. 10.1016/j.pscychresns.2005.06.007 16263248

[76] L. A. Finelli , P. Achermann , and A. A. Borbély , “Individual 'Fingerprints' in Human Sleep EEG Topography,” Neuropsychopharmacology 25, no. 1 (2001): S57–S62. 11682275 10.1016/S0893-133X(01)00320-7

[77] M. Gorgoni , C. Bartolacci , A. D'Atri , et al., “The Spatiotemporal Pattern of the human Electroencephalogram at Sleep Onset after a Period of Prolonged Wakefulness,” Frontiers in Neuroscience 13 (2019): 312. 31001079 10.3389/fnins.2019.00312 PMC6456684

[78] M. Gorgoni , F. Ferlazzo , F. Moroni , et al., “Sleep Deprivation Affects Somatosensory Cortex Excitability as Tested through Median Nerve Stimulation,” Brain Stimulation 7, no. 5 (2014): 732–739. 24953258 10.1016/j.brs.2014.04.006

[79] I. Izquierdo , J. H. Medina , M. Bianchin , et al., “Memory Processing by the Limbic System: Role of Specific Neurotransmitter Systems,” Behavioural Brain Research 58, no. 1‐2 (1993): 91–98. 7907882 10.1016/0166-4328(93)90093-6

[80] R. Joseph , “Environmental Influences on Neural Plasticity, the Limbic System, Emotional Development and Attachment: A Review,” Child Psychiatry and Human Development 29 (1999): 189–208. 10080962 10.1023/a:1022660923605

[81] E. T. Rolls , “Limbic Systems for Emotion and for Memory, but no Single Limbic System,” Cortex; A Journal Devoted to the Study of the Nervous System and Behavior 62 (2015): 119–157. 24439664 10.1016/j.cortex.2013.12.005

[82] M. R. Roxo , P. R. Franceschini , C. Zubaran , F. D. Kleber , and J. W. Sander , “The Limbic System Conception and Its Historical Evolution,” The Scientific World Journal 11, no. 1 (2011): 2427–2440. 10.1100/2011/157150 PMC3236374 22194673

[83] E. T. Bullmore and D. S. Bassett , “Brain Graphs: Graphical Models of the human Brain Connectome,” Annual Review of Clinical Psychology 7, no. 1 (2011): 113–140. 10.1146/annurev-clinpsy-040510-143934 21128784

[84] C. J. V. Stam and E. C. W. Van Straaten , “The Organization of Physiological Brain Networks,” Clinical Neurophysiology 123, no. 6 (2012): 1067–1087. 22356937 10.1016/j.clinph.2012.01.011

[85] C. Civardi , C. Boccagni , R. Vicentini , et al., “Cortical Excitability and Sleep Deprivation: A Transcranial Magnetic Stimulation Study,” Journal of Neurology, Neurosurgery & Psychiatry 71, no. 6 (2001): 809–812. 11723210 10.1136/jnnp.71.6.809 PMC1737655

[86] M. A. Salehinejad , E. Ghanavati , J. Reinders , J. G. Hengstler , M.‐F. Kuo , and M. A. Nitsche , “Sleep‐Dependent Upscaled Excitability, Saturated Neuroplasticity, and Modulated Cognition in the Human Brain,” Elife 11 (2022): 69308. 10.7554/eLife.69308 PMC9225005 35666097

[87] R. Chen , “Studies of Human Motor Physiology with Transcranial Magnetic Stimulation,” Muscle & Nerve 23, no. S9 (2000): S26–S32. 10.1002/1097-4598(2000)999:9<::aid-mus6>3.0.co;2-i 11135281

[88] V. Di Lazzaro , A. Oliviero , M. Meglio , et al., “Direct Demonstration of the Effect of Lorazepam on the Excitability of the human Motor Cortex,” Clinical Neurophysiology 111, no. 5 (2000): 794–799. 10802448 10.1016/s1388-2457(99)00314-4

[89] T. Kujirai , M. D. Caramia , J. C. Rothwell , et al., “Corticocortical Inhibition in Human Motor Cortex,” The Journal of Physiology 471, no. 1 (1993): 501–519. 8120818 10.1113/jphysiol.1993.sp019912 PMC1143973

[90] S. Rossi , M. Hallett , P. M. Rossini , and A. Pascual‐Leone , “Safety, Ethical Considerations, and Application Guidelines for the Use of Transcranial Magnetic Stimulation in Clinical Practice and Research,” Clinical Neurophysiology 120, no. 12 (2009): 2008–2039. 19833552 10.1016/j.clinph.2009.08.016 PMC3260536

[91] H. R. Siebner , T. O. Bergmann , S. Bestmann , et al., “Consensus Paper: Combining Transcranial Stimulation with Neuroimaging,” Brain Stimulation 2, no. 2 (2009): 58–80. 20633405 10.1016/j.brs.2008.11.002

[92] E. V. Orekhova , T. A. Stroganova , J. F. Schneiderman , et al., “Neural Gain Control Measured through Cortical Gamma Oscillations Is Associated with Sensory Sensitivity,” Human Brain Mapping 40, no. 5 (2019): 1583–1593. 30549144 10.1002/hbm.24469 PMC6865508

[93] A. Sanzeni , B. Akitake , H. C. Goldbach , C. E. Leedy , N. Brunel , and M. H. Histed , “Inhibition Stabilization Is a Widespread Property of Cortical Networks,” Elife 9 (2020): 54875. 10.7554/eLife.54875 PMC7324160 32598278

[94] G. Tononi and C. Cirelli , “Sleep Function and Synaptic Homeostasis,” Sleep Medicine Reviews 10, no. 1 (2006): 49–62. 16376591 10.1016/j.smrv.2005.05.002

[95] G. Tononi and C. Cirelli , “Sleep and the Price of Plasticity: From Synaptic and Cellular Homeostasis to Memory Consolidation and Integration,” Neuron 81, no. 1 (2014): 12–34. 24411729 10.1016/j.neuron.2013.12.025 PMC3921176

[96] M. Fukunaga , S. G. Horovitz , P. van Gelderen , et al., “Large‐Amplitude, Spatially Correlated Fluctuations in BOLD fMRI Signals during Extended Rest and Early Sleep Stages,” Magnetic Resonance Imaging 24, no. 8 (2006): 979–992. 16997067 10.1016/j.mri.2006.04.018

[97] H. Helakari , V. Korhonen , S. C. Holst , et al., “Human NREM Sleep Promotes Brain‐Wide Vasomotor and Respiratory Pulsations,” The Journal of Neuroscience 42, no. 12 (2022): 2503–2515. 35135852 10.1523/JNEUROSCI.0934-21.2022 PMC8944230

[98] S. G. Horovitz , M. Fukunaga , J. A. de Zwart , et al., “Low Frequency BOLD Fluctuations during Resting Wakefulness and Light Sleep: A Simultaneous EEG‐fMRI Study,” Human Brain Mapping 29, no. 6 (2008): 671–682. 17598166 10.1002/hbm.20428 PMC6871022

