# Supplementary material for: Inhibitory Decay and Supercritical Brain Dynamics During Sleep Deprivation
Source: Adv Sci (Weinh). 2026 May 15;13(45):e75698. doi: 10.1002/advs.75698 (PMC13336023; doi:10.1002/advs.75698)
Supplement: Supplementary file 1 [file ADVS-13-e75698-s001.docx]

**Comparison models for testing the specificity of inhibitory manipulation**

To examine the specificity of the inhibitory manipulation in the inhibitory-decay cortical network model (IDCNM), we constructed two additional comparison models.

First, we implemented an excitatory enhancement model, in which excitatory efficacy was progressively increased during simulated sleep deprivation by lowering the excitatory firing threshold ($h_{E}$) and increasing excitatory synaptic weights ($W_{\mathrm{EE}}$, $W_{\mathrm{EI}}$) over time. The rates of change of $W_{\mathrm{EE}}$, $W_{\mathrm{EI}}$, and $h_{E}$ during sleep deprivation are symmetrical to the inhibitory parameters in the original model, meaning they have the same numerical values but opposite signs.

$$\begin{aligned} h_{E}\left( t \right)=h_{E}-\theta_{hE}\cdot{SD}_{time}\#\left( 1 \right) \end{aligned}$$

$$\begin{aligned} W_{EE}^{jk}\left( t \right)=W_{IE}^{jk}\cdot\left( 1+\theta_{EE}\cdot{SD}_{time} \right)\#\left( 2 \right) \end{aligned}$$

$$\begin{aligned} W_{EI}^{jk}\left( t \right)=W_{II}^{jk}\cdot\left( 1+\theta_{EI}\cdot{SD}_{time} \right)\#\left( 3 \right) \end{aligned}$$

where $\theta_{hE}=0.01$, $\theta_{EE}=0.01$ and $\theta_{EI}=0.01$.

Second, we implemented a mixed model, in which both excitatory enhancement and inhibitory decay were introduced simultaneously. Specifically, this model combined lowering $h_{E}$and increasing $W_{\mathrm{EE}}$, $W_{\mathrm{EI}}$with raising the inhibitory firing threshold ($h_{I}$) and decreasing inhibitory synaptic weights ($W_{\mathrm{IE}}$, $W_{\mathrm{II}}$).

All models were simulated under the same framework and parameter schedules as the IDCNM, and the resulting criticality-related metrics were computed in the same manner as described above, allowing direct comparison of their temporal trajectories across the sleep deprivation period.

**Specificity of inhibitory decay in reproducing the empirical temporal trajectory**

To test whether the observed dynamics could also be explained by alternative manipulations of network excitability, we compared the original inhibitory-decay model with the excitatory enhancement model and the mixed model.

The results showed that, in both the excitatory enhancement model and the mixed model, the simulated criticality-related metrics exhibited a monotonic increase across the sleep deprivation period (Supplementary Figure 9). In contrast, the empirical data and the inhibitory-decay model both showed a distinct temporal pattern, characterized by an initial increase followed by a relative plateau and partial rebound during the later stage (approximately 24-36 h).

**Control analysis of reversed E/I weight assignment**

To further test whether the model depends on the original assignment of excitatory and inhibitory structure, we performed an additional reversed-weight control analysis. Specifically, we reassigned the initial synaptic weight structure between the two populations, such that the initial excitatory weights were assigned to inhibitory neurons and the initial inhibitory weights were assigned to excitatory neurons, while leaving the remaining model components unchanged. Based on this reassigned architecture, we re-ran the inhibitory-decay model, the excitatory-enhancement model, and the mixed E/I model.

Under this reversed weight assignment, the model no longer showed stable oscillatory activity. Instead, after the first update step (1 ms), the membrane potentials of both excitatory and inhibitory populations rapidly crossed firing threshold and remained above threshold throughout the 1000-ms simulation period. As a result, the network entered a saturated firing state, without the temporal fluctuations observed in the original model, and the derived gamma-band power became degenerate because the population activity no longer contained a meaningful oscillatory structure (Supplementary Figure 10).

By contrast, under the original model configuration, both populations showed stable oscillatory membrane-potential dynamics and yielded interpretable gamma-power estimates. These findings indicate that the model is sensitive to the original E/I assignment, and that the observed sleep deprivation (SD)-like trajectory is not preserved under reversal of the initial E/I weight structure.

**Supplementary Figure 1. The curve of average avalanche size (left panel) and branching ratio (right panel) during sleep deprivation.** Individuals’ scores were showed in gray and averaged responses were plotted in blue. The light blue area represents SEM.

**Supplementary Figure 2.** **Sensitivity analysis using a 4-mm FWHM Gaussian smoothing kernel.** (A) Avalanche distributions at the first time point following preprocessing with a 4-mm smoothing kernel, showing that the data still conformed to a power-law distribution. (B) Longitudinal changes in average avalanche size across the 36-h sleep deprivation period after reanalysis with a 4-mm smoothing kernel. (C) Longitudinal changes in branching ratio across the 36-h sleep deprivation period after reanalysis with a 4-mm smoothing kernel.

**Supplementary Figure 3. Sensitivity analysis using bandpass or high-pass filter.** (upper panel) after preprocessing with a bandpass temporal filter (0.01-0.1Hz), avalanche size distributions at the first time point, longitudinal changes in average avalanche size and branching ratio across the 36-h sleep deprivation. (lower panel) after preprocessing with a high-pass temporal filter (>0.1Hz), avalanche size distributions at the first time point, longitudinal changes in average avalanche size and branching ratio across the 36-h sleep deprivation.

**Supplementary Figure 4. Sensitivity analysis using different BOLD threshold.** (upper panel) at a threshold of 2.0 standard deviations, longitudinal changes in average avalanche size and branching ratio across the 36-h sleep deprivation. (lower panel) at a threshold of 2.2 standard deviations, longitudinal changes in average avalanche size and branching ratio across the 36-h sleep deprivation.

**Supplementary Figure 5. Changes in avalanche dynamics parameters of DAN, VAN, FPN, and DMN networks during sleep deprivation.** (A-B) The dynamic curves of the average avalanche size and branching ratio of DAN during the SD period. The p-value was showed above the curve. The case where the branching ratio is equal to 1 is shown by a dotted line, indicating the standard critical state. (C) The distribution of correlation coefficients between the average avalanche size and branching ratio of DAN and the sleep pressure. Equivalent to the formats of (A, B), the dynamic curves of the average avalanche size and branching ratio of VN (D, E), FPN (G, H) and DMN (J, K) were shown. Equivalent to the formats of (C), the distribution of correlation coefficients between individuals’ sleep pressure and the average avalanche size and branching ratio of VN (F), FPN (I) and DMN (L) were shown.

**Supplementary Figure 6. The dynamic curves of the average avalanche size and branching ratio of VN, SMN and LN during the SD period under a global BOLD threshold.** Considering the threshold ranges of different brain networks, the global threshold is set to 1.3 times the standard deviation. The p-value was showed above the curve.

**Supplementary Figure 7. Results of post-hoc tests of low-degree node ratio and high-degree node ratio.** For post-hoc results, red indicates an increase in the behavioral measurement value at the second time point compared to the measurement value at the first time point, and blue indicates a decrease in the measurement value. Asterisks indicate significant difference at *p*<0.05 (FDR corrected).

**Supplementary Figure 8. The dynamic curves of low-degree node ratio and high-degree node ratio with different parameter combinations during the SD period are shown.** We employed two parameter combinations. First, we varied the FC threshold across a reasonable range (*r* > 0.2, 0.25, 0.35, and 0.4), while keeping the definitions of low-degree and high-degree nodes unchanged (< 30 and > 300, respectively). Second, we fixed the FC threshold at *r* > 0.3 and varied the degree cutoffs used to define low-degree and high-degree nodes (degree < 10 and > 100, < 20 and > 200, < 40 and > 400, and < 50 and > 500). Across both sets of analyses, the same overall pattern was consistently observed: with prolonged wakefulness, the proportion of high-degree nodes increased, whereas the proportion of low-degree nodes decreased.

**Supplementary Figure 9. Excitatory enhancement model and mixed model.** First, we implemented an excitatory enhancement model, in which excitatory efficacy was progressively increased during simulated sleep deprivation by lowering the excitatory firing threshold ($h_{E}$) and increasing excitatory synaptic weights ($W_{\mathrm{EE}}$, $W_{\mathrm{EI}}$) over time. The rates of change of $W_{\mathrm{EE}}$, $W_{\mathrm{EI}}$, and $h_{E}$ during sleep deprivation are symmetrical to the inhibitory parameters in the original model, meaning they have the same numerical values but opposite signs. Second, we implemented a mixed model, in which both excitatory enhancement and inhibitory decay were introduced simultaneously. Specifically, this model combined lowering $h_{E}$and increasing $W_{\mathrm{EE}}$, $W_{\mathrm{EI}}$with raising the inhibitory firing threshold ($h_{I}$) and decreasing inhibitory synaptic weights ($W_{\mathrm{IE}}$, $W_{\mathrm{II}}$). Whether using dynamic curves or post-hoc analysis, the simulated criticality-related metrics exhibited a monotonic increase in both the excitatory enhancement model and the mixed model, across the sleep deprivation period. Data are shown as mean ± SEM.

**Supplementary Figure 10. Inhibitory decay model (A-C), Symmetric model (D-F) and Mixed model (G-I) that reassign the initial weights of E/I.** Reversed E/I weight assignment abolishes stable oscillatory dynamics and produces degenerate gamma estimates across the inhibitory-decay, excitatory-enhancement, and mixed E/I simulations.

**Supplementary Table 1.** The optimal BOLD thresholds for each functional network of Yeo 7-atlas. The corresponding power-law exponents and branching ratios are also shown.

|  | VN | SMN | DAN | VAN | LN | FPN | DMN |
| --- | --- | --- | --- | --- | --- | --- | --- |
| Optimal thresholds | 1.6*SD | 1.4*SD | 1.6*SD | 1.4*SD | 1.3*SD | 1.6*SD | 1.7*SD |
| Power-law exponents | -1.535 | -1.520 | -1.508 | -1.477 | -1.635 | -1.503 | -1.476 |
| Branching ratios | 0.963 | 1.015 | 0.965 | 1.035 | 0.952 | 0.958 | 0.942 |
